# The genome of *Onchocerca volvulus*, agent of river blindness

**DOI:** 10.1038/nmicrobiol.2016.216

**Published:** 2016-11-21

**Authors:** James A. Cotton, Sasisekhar Bennuru, Alexandra Grote, Bhavana Harsha, Alan Tracey, Robin Beech, Stephen R. Doyle, Matthew Dunn, Julie C. Dunning Hotopp, Nancy Holroyd, Taisei Kikuchi, Olivia Lambert, Amruta Mhashilkar, Prudence Mutowo, Nirvana Nursimulu, Jose M. C. Ribeiro, Matthew B. Rogers, Eleanor Stanley, Lakshmipuram S. Swapna, Isheng J. Tsai, Thomas R. Unnasch, Denis Voronin, John Parkinson, Thomas B. Nutman, Elodie Ghedin, Matthew Berriman, Sara Lustigman

**Affiliations:** 1 Wellcome Trust Sanger Institute, Wellcome Genome Campus, Hinxton, Cambridgeshire CB10 1SA, UK; 2 Laboratory of Parasitic Diseases, National Institute of Allergy and Infectious Diseases, NIH, Bethesda, Maryland 20892, USA; 3 Center for Genomics and Systems Biology, Department of Biology, New York University, New York, New York 10003, USA; 4 Institute of Parasitology, McGill University, Montreal, Quebec H9X 3V9, Canada; 5 Institute for Genome Sciences, Department of Microbiology and Immunology, University of Maryland School of Medicine, Baltimore, Maryland 21201, USA; 6 Global Health Infectious Disease Research Program, Department of Global Health, College of Public Health, University of South Florida, Tampa, Florida 33612, USA; 7 European Molecular Biology Laboratory, European Bioinformatics Institute, Wellcome Genome Campus, Hinxton, Cambridgeshire CB10 1SD, UK; 8 Department of Computer Science, University of Toronto, Toronto M5S 3G4, Canada; 9 Division of Molecular Structure and Function, Research Institute, Hospital for Sick Children, Toronto, Ontario M5G 1X8, Canada; 10 Laboratory of Malaria and Vector Research, National Institute of Allergy and Infectious Diseases, NIH, Bethesda, Maryland 20892, USA; 11 Children’s Hospital of Pittsburgh, University of Pittsburgh School of Medicine, Pittsburgh, Pennsylvania 15224, USA; 12 New York Blood Center, New York, New York 10065, USA; 13 Departments of Biochemistry and Molecular Genetics, University of Toronto, M5S 1A8, Canada; 14 College of Global Public Health, New York University, New York, New York 10003, USA

## Abstract

Human onchocerciasis is a serious neglected tropical disease caused by the filarial nematode *Onchocerca volvulus* that can lead to blindness and chronic disability. Control of the disease relies largely on mass administration of a single drug, and the development of new drugs and vaccines depends on a better knowledge of parasite biology. Here, we describe the chromosomes of *O. volvulus* and its *Wolbachia* endosymbiont. We provide the highest-quality sequence assembly for any parasitic nematode to date, giving a glimpse into the evolution of filarial parasite chromosomes and proteomes. This resource was used to investigate gene families with key functions that could be potentially exploited as targets for future drugs. Using metabolic reconstruction of the nematode and its endosymbiont, we identified enzymes that are likely to be essential for *O. volvulus* viability. In addition, we have generated a list of proteins that could be targeted by Federal-Drug-Agency-approved but repurposed drugs, providing starting points for anti-onchocerciasis drug development.

The filaria are a group of tissue-dwelling parasitic nematodes of vertebrates that are spread by blood-feeding arthropods. *Onchocerca volvulus* is the most pathogenic and is the agent of onchocerciasis (or ‘river blindness’), a leading cause of morbidity and socioeconomic loss for the world’s poorest populations^1^. Approximately 17 million people are still infected with *O. volvulus*, predominantly in Africa^2^. Infections are chronic and manifest clinically as debilitating skin disease and—in 1.2 million people—vision impairment or blindness. First-stage larvae, known as microfilariae (L1/mf), are produced by fertile female worms residing within onchocercomata (nodules). They migrate to the skin and other organs (for example, the anterior chamber of the eye), where they induce inflammatory reactions that are responsible for most *Onchocerca*-related pathology.

Onchocerciasis was identified by the World Health Organization (WHO) as a potential candidate for disease elimination through annual (or semiannual) mass drug administration (MDA) of ivermectin^3^, an approach that has eliminated onchocerciasis from all but two countries in the Americas^4^. Ivermectin is solely microfilaricidal, which means it must be given over decades, past the lifespan of the long-lived adult worms^5,6^. Moreover, in much of Central Africa where *Loa loa* is co-endemic with *O. volvulus*, ivermectin cannot be used due to the risk of *Loa*-associated irreversible neurological severe adverse events and death^7^. Despite its success in Latin America and small foci in Africa, elimination of onchocerciasis in Africa is unlikely to be achieved within the proposed timeframes solely through MDA with ivermectin^8^. Reliance on a single drug also increases the potential for the emergence of ivermectin-resistant *O. volvulus*^5^, making the development of new drugs or novel therapies imperative.

To gain better insight into this important but neglected pathogen, we generated a high-quality genome assembly of *O. volvulus*. Although draft whole-genome assemblies exist for other filarial nematode species^9–13^, we have, for the first time in any species of this group, reconstructed whole chromosomes, including a sex chromosome. We also generated a genome assembly for its obligate intracellular endosymbiont *Wolbachia* (*w*Ov) and transcriptional data from eight life stages. Our analysis highlights the metabolic interplay between *O. volvulus* and *w*Ov as a path to novel drug targets.

## Results

### *O. volvulus* genome structure and features

**Figure 1:**
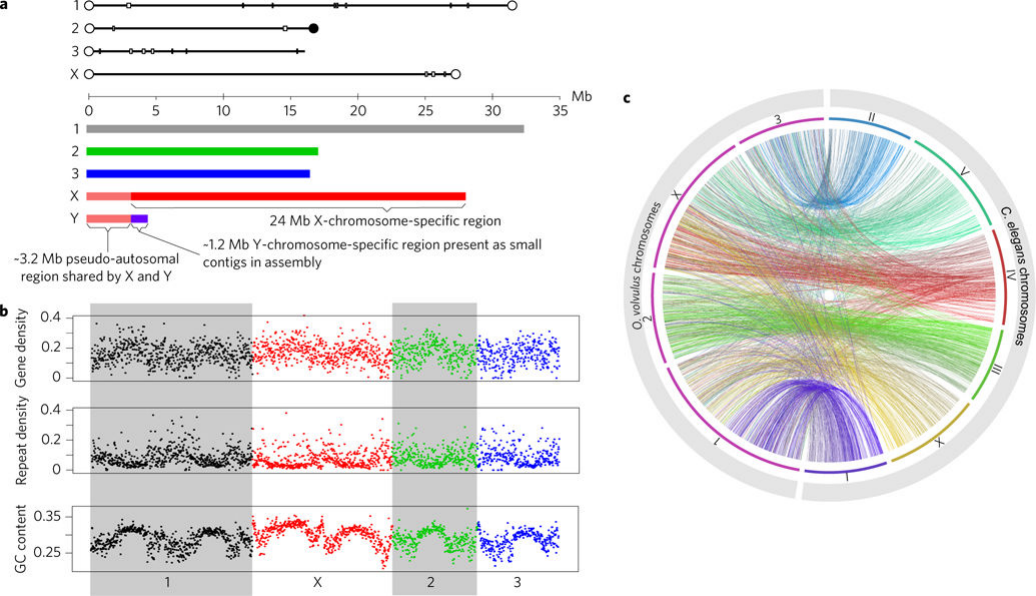
*O. volvulus* chromosomes **a**, Lines above the axis show sizes of *O. volvulus* chromosomes in the assembly and the locations of potential telomeric repeats. Filled circles indicate telomere repeats present in the assembly, open circles are ends of optical map scaffolds (Supplementary Fig. 1c). Rectangular boxes/bars indicate sequence gaps of at least 50 kb. The inferred karyotype for *O. volvulus* is shown below the axis, based on the assembly and sequence coverage data. **b**, GC content, gene density and repeat density (proportion of bases in each window covered by genes/at least one annotated repeat) in non-overlapping 10 kb windows for each of the four large scaffolds. Colours and shading indicate scaffold boundaries. **c**, Comparison of four *O. volvulus* chromosomes with six *C. elegans* chromosomes. Links show PROmer hits with similarity greater than 70% over at least 100 amino acids, coloured according to chromosome location of *C. elegans* hit.

The 97 Mb nuclear genome of *O. volvulus* comprising three autosomes and a pair of sex chromosomes^14^ was assembled using a combination of sequencing, an optical map and manual improvement. Four large scaffolds (16–31 Mb) comprise 94% of the assembly and seven out of eight of their ends correspond to ends of optical maps or telomeric repeats (Fig. 1a and Supplementary Fig. 1). These scaffolds thus represent essentially complete chromosomes of *O. volvulus*. This is the highest-quality assembly for any parasitic nematode (Supplementary Tables 1 and 2) and only the fourth nematode species^15–17^ for which chromosome sequences are available. The assembly also includes the mitochondrial genome and complete *w*Ov genome.

By analysing sequence data from male and female worms, we identified scaffold OM2 as the X chromosome (Supplementary Fig. 2a). For a long contiguous portion (22.2 of 25.5 Mb of the scaffold) the median depth of coverage of male sequence data was 50% that for the rest of the genome. The same region had a coverage of 75% using data from mature females, which are likely to be gravid and contain a mixture of male and female cells, and 100% using data from juvenile female worms only (Supplementary Fig. 2a and Supplementary Table 3). We propose that this region represents the X-chromosome-specific sequence, while the rest of the scaffold is a 3.2 Mb pseudo-autosomal region (PAR) shared by X and Y chromosomes and presumably still capable of chromosomal crossover. Other scaffolds lack data from juvenile females and show low coverage in adult female libraries (Supplementary Fig. 2c,d), allowing us to identify ∼1.2 Mb as the potentially Y-specific sequence. Only this small portion of the Y chromosome is present in our assembly as this chromosome is largely pseudo-autosomal (Fig. 1a) and so mostly assembled with the X chromosome. The small extent of sequence divergence between X and Y and the presence of an extensive PAR confirm that this evolved recently from an ancestral XO karyotype^14^ and contrasts with the situation in other nematodes that have X and Y chromosomes, where the Y is largely unique, repeat-rich and degenerate^18^. Furthermore, one region of the PAR adjacent to the X-specific region shows an excess of heterozygous sites missing in the juvenile female sample (Supplementary Fig. 2b). This represents a region in which X and Y have begun to diverge, but where the two chromosomes are sufficiently similar that they are still represented by the same region of the assembly. We propose that this is a region of more recent divergence between X and Y chromosomes than the X- and Y-specific regions. This suggests a process of sex chromosome evolution similar to that observed in other systems, in which recombination suppression and subsequent divergence between sex chromosomes occurs in a patchy way, leading to different ‘strata’ of divergence^19^.

We identified over 97% of a conserved set of eukaryotic genes^20^ in this assembly, with five of the six missing genes also missing from all other filarial genome assemblies, presumably reflecting ancestral gene losses from this group (Supplementary Table 4). *O. volvulus* shows large-scale variation in gene density, GC content and repeat density (Fig. 1b and Supplementary Table 5), establishing that these patterns are present in nematodes beyond *Caenorhabditis* spp. The pattern of variation on chromosomes 1 and X is more complex, with two peaks in gene density and GC. We propose that this pattern is due to the origins of these chromosomes as fusions of two ancestral chromosomes. For comparison of gene content, we have also generated sequence data for the related species *O. ochengi*, a filarial parasite of cattle, and produced a fragmented draft assembly of this species (Supplementary Tables 1 and 2).

### Gene content

A total of 12,143 protein-coding genes were predicted in the *O. volvulus* genome guided by RNAseq data from eight stages of the parasite life cycle (Supplementary Tables 1 and 2). The majority (∼91%) of genes had orthologues in other nematodes, with ∼9% (1,173) being *O. volvulus*-specific, with little or no homology to genes annotated in other helminths (Supplementary Table 6). Predicted proteins were classified into functional categories^21^ (Supplementary Fig. 3a), although 44% of *O. volvulus* proteins have no predicted function. The distinctive biology of *O. volvulus* is likely to be underpinned by genes with potentially novel functions and with relatively few homologues in other helminth parasites. Of the *O. volvulus*-specific genes, 92% encode putative proteins of unknown function, of which 7% are potentially secreted (Supplementary Fig. 3b and Supplementary Table 6).

A total of 3,152 protein-coding genes were present on the large contiguous X-chromosome-specific region and an additional 113 on small contigs assigned to X, while only 100 genes (Supplementary Table 7) were inferred to be on Y-chromosome-specific contigs. The X-specific genes are enriched for those encoding putative G-protein coupled receptors, translational release factors, calcium-binding proteins and membrane proteins involved in cell–cell communication.

### Gene synteny and gene family expansions

The free-living nematode *Caenorhabditis elegans* remains the most genetically tractable model nematode, so we used that species to perform comparative analyses with *O. volvulus*. Mapping orthologues indicated a clear correspondence between *C. elegans* chromosomes I, II and III with *O. volvulus* chromosomes 1, 3 and 2, respectively. *C. elegans* chromosomes IV and V orthologues match mostly the *O. volvulus* X chromosome, while *C. elegans* chromosome X orthologues are split between *O. volvulus* chromosomes 1 and X (Fig. 1c). Gene synteny between *O. volvulus* chromosomes and the ten largest *O. ochengi* scaffolds shows that both genomes are predominantly co-linear, but not all genes have predicted one-to-one orthologues (Supplementary Fig. 4).

**Figure 2:**
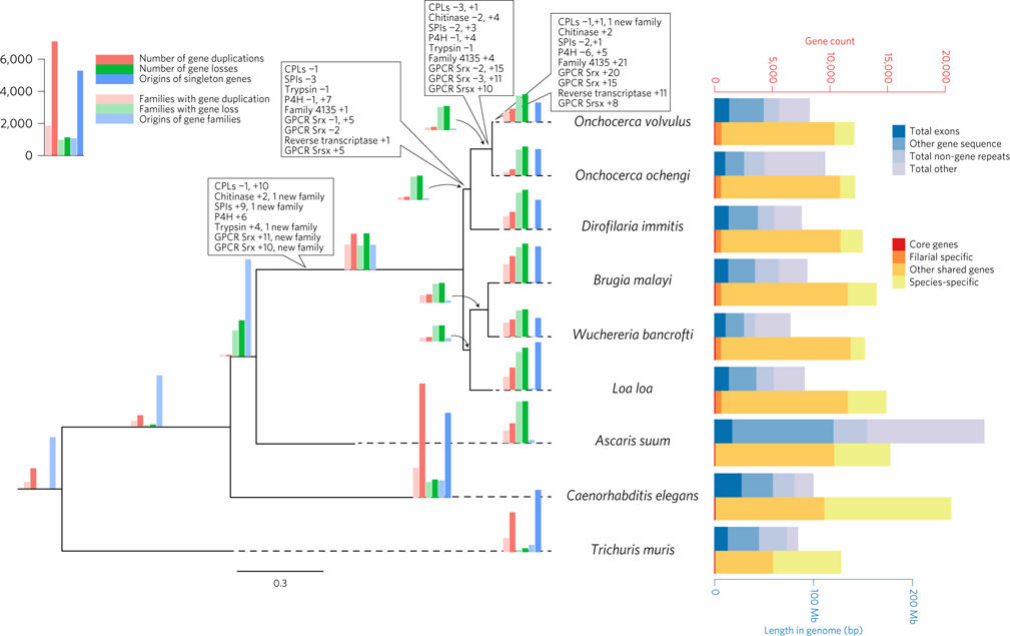
Gene family evolution and comparative genomics of *O. volvulus* and relatives Maximum-likelihood genome phylogeny of six filarial nematode species and three outgroup species. All nodes were fully supported by 100 bootstrap replicates. The phylogeny is annotated with histograms showing the number of duplications (red) and losses (green) for individual genes (dark red or dark green); number of families (light red or light green) with one or more duplications/losses; and numbers of gene families (light blue) inferred to appear on each branch and (on terminal branches) numbers of singleton genes (dark blue) as estimated by the Ensembl Compara pipeline. Note that our data cannot reconstruct gene losses on the most basal branch of the tree. Bar charts in yellow-red summarize the evolutionary history of the genome of each species, defining genes shared among all nine nematode species, the six filarial species and genes with more complex patterns of conservation. The total heights of these bars represent the total number of protein-coding loci annotated on each genome. Boxes on branches show numbers of gene duplications (+X) and losses (−X) in five gene families of specific interest in *O. volvulus*: trypsin, cathepsin-L like proteases (CPL), chitinase, serine protease inhibitors (SPI) and prolyl-4 hydroxylase alpha-subunits (P4H) and in other families with many gene duplications on the branch leading to *O. volvulus*. Duplications are observed in two different Srx GPCR families; the reverse transcriptase gene family could be missing from some species because of differences in annotation and repeat finding methods. Family 4135 comprises weakly conserved hypothetical proteins. Stacked bar charts in blue summarize the genome of each species, with total heights representing the size of each genome assembly, divided into exons, other genic sequences (introns and UTRs; non-coding genes), annotated repeats and DNA sequence not annotated in any of these categories.

We further investigated the evolutionary history of *O. volvulus* genes using Ensembl Compara^22^. This produced high support for the traditional classification of this group as found in previous molecular work^23^ and confirmed the close relationship between *O. volvulus* and *O. ochengi* (Fig. 2). We observed many gene duplication events of potential biological importance in the evolutionary history of *O. volvulus* (Supplementary Table 8 and Supplementary Data). For example, the gene family encoding the α subunit of the enzyme collagen prolyl 4-hydroxylase (C-4PHα), an important enzyme for collagen synthesis in the cuticle of nematodes, has been repeatedly duplicated in the lineage leading to *O. volvulus*, resulting in a paralogous family of 16 genes. Two copies are clearly truncated and may be pseudogenes, while at least 11 of these paralogues are full-length, have the conserved catalytic sites residues^24^ (Supplementary Fig. 5) and are predicted to be secreted. This drastic expansion probably reflects the requirement for changes in collagen composition during cuticle remodelling throughout *O. volvulus* development.

**Figure 3:**
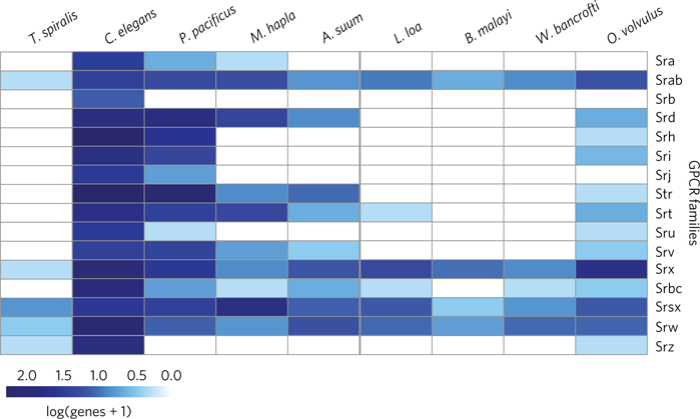
GPCRs Profile of GPCR families present in nematode genomes in comparison to *O. volvulus*. The log of the number of genes plus one is plotted per family.

Similarly, three G-protein-coupled receptor (GPCR) gene families show a remarkable expansion in *O. volvulus* (Fig. 2 and Supplementary Table 8). In general, filarial genomes lack several GPCR gene families present in *C. elegans*, including Srj, Sra and Srb receptors^9^. However, unlike the other filariae, *O. volvulus* contains members of all other GPCR families represented in *C. elegans* (Fig. 3). For example, it retains the Str family involved in odorant detection, a family not found in other filariae but present in *Ascaris suum*, and the root-knot nematode *Meloidogyne hapla*, perhaps reflecting responses to specific cues in their environments^9^. The maintenance of Str and other families of GPCRs may suggest their importance throughout the developmental stages of *O. volvulus*, but could also reflect the high quality of the sequence assembly and annotation. Further underlining the importance of GPCRs to *O. volvulus* biology, the Srx and Srsx families are among the most duplicated genes in the *O. volvulus* genome, most striking of which is the expansion of the Srx family, with 35 duplications inferred to be specific to *O. volvulus*.

In contrast to GPCRs, the nuclear hormone receptor (NHR) gene family is particularly sparse. NHRs are important transcriptional modulators that regulate cellular differentiation, homeostasis and reproduction in nematodes and other animals^25^. *C. elegans* has over 270 NHRs—the highest number identified in any species—but the filarial nematodes have a far smaller repertoire. *Brugia malayi* has 50 and *O. volvulus* even fewer, with only 24 classical NHRs and 8 orphan receptors identified (Supplementary Table 9 and Supplementary Fig. 6). Despite this, *O. volvulus* has orthologues of all five *C. elegans* NHR genes that exclusively participate in moulting and metamorphosis^26,27^ (Supplementary Table 10). Interestingly, the ecdysone receptor (OVOC9104) was gained at the base of the filarial lineage. Ecdysone and related hormones regulate moulting and metamorphosis in arthropods, so it is tempting to speculate that this gain in NHRs may be an adaptation to the insect host.

Control of the neuromusculature is a principal target for many anthelmintic drugs, where they bind and activate the pentameric ligand-gated ion channels (pLGICs) that mediate fast-synaptic signalling. Like other filarial nematodes, *O. volvulus* encodes fewer pLGIC subunit genes (only 48 compared to 120 in *C. elegans*^28^ and other clade V nematodes^29^), but has retained genes encoding subunits of the glutamate- and GABA-gated chloride channels (Supplementary Fig. 7) that are putative targets for ivermectin^30^. In filarial nematodes, ivermectin paralyses or kills microfilariae gradually, but also suppresses microfilarial production from adult females. *O. volvulus* encodes an orthologue of the *avr-14* ivermectin target that in *B. malayi* is expressed within reproductive tissue; this may explain the effect of ivermectin on microfilarial production^31^.

Although the emergence of drug resistance in human parasites has been less rapid than in parasitic nematodes of livestock, cases of unexpected earlier reappearance of *O. volvulus* microfilariae following ivermectin treatment have been reported^31^. The mechanisms of ivermectin resistance remain unclear, but polymorphism of P-glycoprotein (Pgp) drug transporters in other nematodes has been linked to resistance, together with evidence that Pgp inhibitors can increase drug efficacy^32,33^. The Pgps are members of the ATP-binding cassette (ABC) transporter family. The genome of *O. volvulus* contains relatively few ABC transporter orthologues, with only 10 compared to 54 in *C. elegans*^31^.

### Metabolic reconstruction and identification of potential drug targets

To examine the metabolic capabilities of *O. volvulus* and *L. loa*, a filarial parasite without a *Wolbachia* endosymbiont, we performed genome-scale metabolic reconstructions (Supplementary Tables 11–13). The resultant networks comprise 767 reactions (378 distinct enzymes) for *O. volvulus* and 648 reactions (301 enzymes) for *L. loa*. Each share a core of 628 reactions, with the majority (100 of 139) of additional reactions in the *O. volvulus* reconstruction contributed by *w*Ov. Next, we performed flux balance analysis (FBA)^34,35^ to investigate the impact of single reaction knockouts on parasite growth. Metabolites were made freely available across reactions, an assumption that will minimize false-positive essential reaction predictions at the expense of false-negative predictions (for details see Methods).

For *O. volvulus*, FBA predicted 71 essential reactions (Table 1 and Supplementary Tables 11 and 12). For *L. loa*, 112 reactions were predicted to be essential (70 common to *O. volvulus* essential reactions), including 23 transport reactions (Supplementary Table 12). Essential reactions are associated with nucleotide and lipid metabolism, energy production, biosynthesis of cofactors and transport (Table 1). Only threonine transport was essential for *O. volvulus* but not *L. loa*, which can produce its own threonine. In contrast, *O. volvulus* benefits from possible *w*Ov contributions to fatty acid metabolism, haem synthesis and nucleotide metabolism, which, unlike *L. loa*, does not require the salvage of key metabolites in these pathways from the host. The endosymbiont also allows the conversion of lysine from aspartate (Supplementary Table 11). Finally, a single reaction predicted to be essential to both parasites is uniquely provided by *w*Ov: NAD kinase (Enzyme Commission number: EC 2.7.1.23; Table 1 and Supplementary Table 11).

**Figure 4:**
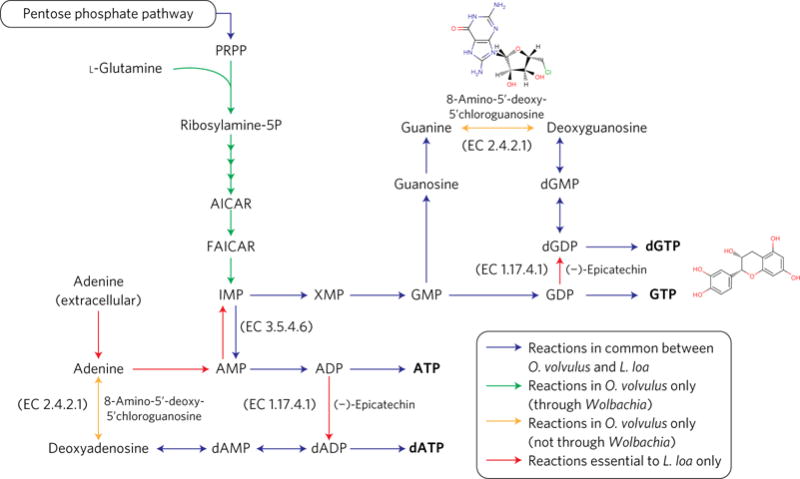
Contribution of *Wolbachia to O. volvulus* in purine metabolism Inhibitors 8-amino-5′-deoxy-5′chloroguanosine and (−)-epicatechin (shown in the figure) have been found against the activities of purine nucleoside phosphorylase (EC 2.4.2.1) and ribonucleoside diphosphate reductase (EC 1.17.4.1)^113,114^. The chemical structures of the inhibitors shown were obtained from the Braunschweig Enzyme Database (BRENDA)^115^. Metabolites in bold represent biomass components.

Focusing on purine metabolism (Fig. 4), *wOv* provides an alternative route for inosine monophosphate (IMP) production in *O. volvulus*, a crucial precursor in purine metabolism, while *L. loa* depends exclusively on adenine import. It is not known whether IMP produced by *w*Ov is available to the nematode, but given their ability to take up purines and purine nucleosides^36,37^, the relative susceptibility of the two species to the inhibition of adenosine monophosphate (AMP) aminohydrolase (EC 3.5.4.6) activity warrants further investigation. Furthermore, the presence of purine-nucleoside phosphorylase (EC 2.4.2.1)—enabling the conversions of guanine and adenine to deoxyguanosine and deoxyadenosine, respectively—ensures *O. volvulus* is robust to the deletion of ribonucleoside-diphosphate reductase (EC 1.17.4.1). Given the strikingly different pathways involved in the production of purines for the two species, any reliance by *O. volvulus* on this alternative pathway could be exploited to selectively target *O. volvulus*^7^, a credible prospect because inhibitors of purine-nucleoside phosphorylase already exist^38^.

### Insights into possible repurposed drugs or new drug targets

We have also investigated potential *O. volvulus* targets of currently available Federal Drug Agency (FDA)-approved drugs. We excluded *w*Ov loci, as repurposing of antibacterial compounds for anti-*Wolbachia* chemotherapy has been extensively investigated^39^. This strategy identified 51 *O. volvulus* proteins, mostly enzymes and proteins involved in ion transport and neurotransmission, that 85 drugs may target (Supplementary Table 14). We used the WHO Anatomical Therapeutic Classification (ATC) to remove from the set those drugs classed as anti-neoplastics (these are likely to have intolerable side effects for anthelmintic use), resulting in a set of 42 drugs. By screening these targets based on sequence features, we identified 16 *O. volvulus* proteins likely to be good drug targets (Table 2).

In a second approach, we leveraged known mechanisms of action of particular drug classes on specific nematode protein families to prioritize from a wider set of potential targets. Among the *O. volvulus* protein families with at least one drug target, those comprising zinc finger C4, NHR, dopamine neurotransmitter, GPCR, serotonin receptors, GABA receptor and ion channels could be considered ‘privileged’ based on the extensive list of drugs by which they may be targeted. The genome sequence reveals the full repertoire of these families, providing a rich resource of potential leads for future drug development efforts (Supplementary Fig. 8). Finally, there is the possibility that existing antiparasitic drugs such as levamisole and morantel, which each target distinct classes of acetylcholine-gated cation channels^40^ (AcHR), should be revisited as antifilarial agents. Levamisole targets *unc*-38 and *unc*-63 (duplicated in *O. volvulus*), while morantel targets *acr-26* and *acr-27* (OVOC7603; a distinct class of AcHR found only in parasitic nematodes). Importantly, these targets are all conserved between *O. volvulus* and *B. malayi*^41,42^. The availability of the complete genome sequence will enhance the opportunity to investigate the mechanisms of action of existing classes of anthelminthics.

### *w*O*v* and lateral gene transfer

In addition to the filarial genome, we assembled the complete 956 kb genome of *w*Ov (Supplementary Table 15). The *O. volvulus* genome was examined for evidence of lateral gene transfers (LGTs) from *w*Ov to its host. These are termed nuclear *Wolbachia* transfers or nuwts. In total, 531 putative nuwts were identified. Interestingly, one LGT event (OVOC_OM2 22,596,036–22,595,692) was found to have a best match to *Midichloria mitochondrii*, the endosymbiont of tick mitochondria (Supplementary Table 16). Seven nuwts were larger than 1 kb (maximum of 8.1 kb), four of which were confirmed by amplification, cloning and sequence verification of the *Wolbachia*–nematode junction (Supplementary Table 17). In a gene-based analysis aimed at detecting more divergent nuwts, a majority (98%) of the 576 regions that contain nuwt open reading frames were fragmented, but four were full-length, potentially functional genes, with relatively few overall mutations, suggesting they may be full-length merely because they are recent transfers (Supplementary Table 16). Overall, nuwts appear to be largely nonfunctional in this genome.

### Insights into host–parasite interactions

*O. volvulus*, like the other filarial parasites, interacts with its definitive human host and its intermediate arthropod host (*Simulium* spp.) during its life cycle. These parasites must have their own innate immune system to protect them from microbial pathogens, but they also are thought to have evolved mechanisms to subvert both human and insect host defence mechanisms. Although molecules such as immunoglobulins or Toll-like receptors (TLRs) are absent in all filarial nematodes, *O. volvulus* and other filariae sequenced to date have homologues of some downstream proteins from the TLR signalling pathway (Supplementary Table 18). The innate immune system encoded by the *O. volvulus* genome also includes C-type lectins, galectins, jacalins and scavenger receptors. Similar to the other filariae, *O. volvulus* does not appear to produce the antibacterial peptides seen in *C. elegans* (and in other non-filarial nematodes), although it is possible that different peptides are produced by filariae.

Analysis of the putative proteome of *O. volvulus* identified a number of human cytokine and chemokine mimics and/or antagonists (Supplementary Table 18). In addition, the *O. volvulus* genome encodes 12 serine protease inhibitors (SPIs) including serpins and small SPIs, and five cysteine protease inhibitors including cystatins. Both protein families have been shown to interfere with antigen processing and presentation^43^ and are potentially involved in immune regulation and in parasite interference with the host immune response^44^. The *O. volvulus* genome also encodes proteins with sequences similar to those of human autoantigens, some of which have been implicated in inducing cross-reactive antibodies that have been connected with the pathogenesis of posterior eye disease^45^ and the nodding syndrome^46^.

## Discussion

The *O. volvulus* genome assembly represents the highest-quality genomic data available for any non-model nematode species. It will be a critical resource for research on other filarial nematodes that reside in different niches within the host, for which only draft genomes are available. Comparative analysis with other nematode genomes has confirmed the recent evolution of an XY karyotype suggesting ancient chromosomal fusions led to the formation of the *Onchocerca* chromosomes. It should be possible to confirm this hypothesis when chromosome-scale assemblies for filarial nematodes without these fusions become available.

The genome data constitute an invaluable and comprehensive resource for the development of new and urgently needed interventions against onchocerciasis and other filariases. In particular, we describe the orthologues and paralogues of known or suspected targets of existing anthelmintic compounds and identify targets of other licensed compounds that could show activity against *O. volvulus*. Our analysis of the targets of these drugs highlights several protein families that could guide further drug discovery in *Onchocerca*. Finally, we performed metabolic reconstructions of *O. volvulus* in conjunction with its *Wolbachia*. By investigating *in silico* the impact of single reaction knockouts on parasite growth, we identified enzymes likely to be essential to *O. volvulus* viability. While an ideal method would model the multicellular nature of *O. volvulus* and treat *Wolbachia* and *Onchocerca* metabolisms as two separate compartments with transport processes between them, we believe our approach sets a basis for future methodologies to understanding filarial metabolism and approaches the current state of the art in *C. elegans* metabolic models^47^. By comparing *O. volvulus* and *L. loa*, which does not harbour *Wolbachia*, we also identified a unique *O. volvulus* target and those that are compensated by symbiosis. Developing additional therapeutic strategies seems likely to be vital in achieving timely elimination of onchocerciasis^8^, and these targets are now important candidates for experimental testing and validation.

## Methods

### Parasite material for genome sequencing

All *O. volvulus* parasite material used for genome sequencing was collected in the research facility at the Tropical Medicine Research Station, Kumba, Cameroon. Written informed consent was obtained. In cases of illiteracy, a literate witness signed and a thumbprint was made by the participant. Institutional Review Board (IRB) approvals were obtained from both the New York Blood Center and from the Tropical Medicine Research Station, Kumba (Protocols 321 and 01, respectively). The individuals who consented to participate in the study were born or had resided for more than ten years in villages around Kumba. They were confirmed to have microfilariae in their skin snips and clinical symptoms of disease, such as dermatitis, nodules and ocular lesions. None of the subjects had received ivermectin treatment before the study. The adult worm samples were obtained as part of a nodulectomy campaign conducted in villages surrounding Kumba in 1996–1998 and in 2006. Nodules were excised under sterile conditions and were treated with collagenase overnight, following the protocol of Schulz-Key and colleagues^48^. Briefly, cleaned individual nodules were immersed in 0.5% collagenase (Sigma grade IV) in RPMI 1640 containing 10% FCS + 200 units of penicillin and 200 μg ml^−1^ streptomycin. The flat tubes containing the nodule were then placed in a rocking water bath and incubated at 35 °C until the tissue was completely digested. Once digested, the liberated worms were unravelled from residual tissue with mounted needles under a dissecting scope and then washed in several changes of RPMI. Individual female worms were snap-frozen in Eppendorf tubes with liquid nitrogen. They were then stored and shipped in liquid nitrogen and, upon arrival in New York, stored at −80 °C until shipment on dry ice to the Wellcome Trust Sanger Institute.

Freshly dissected *O. volvulus* L3s were also cryopreserved according to the method described by Cupp and colleagues^49^ and were shipped to the New York Blood Center in liquid nitrogen and, upon arrival in New York, were stored in liquid nitrogen. To collect sexed juvenile adult worms (40 days in culture), thawed and washed L3s were cultured for 14 days in the presence of peripheral blood mononuclear cells (PBMCs) (1.5 × 10^5^ per well of a 96-well plate) and then over a monolayer of human dermal fibroblasts (10^5^ per well of a 24-well plate) (S. Lustigman, unpublished data). On day 40, they were separated into juvenile female and male worms based on their size (males being much smaller than females) and the morphology of the posterior end^50^. They were then frozen individually, stored at −80 °C until shipment on dry ice to the Wellcome Trust Sanger Institute.

Cows that had grazed in northern Cameroon, where *O. ochengi* is highly endemic, were brought to abattoirs located in Douala, Cameroon. Subcutaneous nodules containing adult *O. ochengi* worms were identified on the umbilical skin of slaughtered infected cows. Adult worm masses containing one viable adult female and zero to several adult males were then carefully recovered from the purchased skins by dissection of the nodule with a sterile razor blade and then snap-frozen in liquid nitrogen. The material was transported to the USA in liquid nitrogen and, upon arrival in New York, stored at −80 °C.

### Parasite material for transcriptomics

All parasite material was prepared in the Tropical Medicine Research Station, Kumba, Cameroon, between 1993 and 1999, except for female worm samples, which were from both Cameroon and Ecuador, where samples were collected as part of a previous study^51^. L3 were obtained from *Simulium damnosum* flies 7 days after infection with skin microfilariae, as described previously^52,53^. To obtain moulting larvae, freshly dissected L3s were cultured *in vitro* in groups of ten larvae in 96-well plates containing a 1:1 mixture of Iscove’s modified Dulbecco’s medium and NCTC-135, 20% fetal calf serum and antibiotic-antimycotic solution (Life Technologies) for 3 days at 37 °C. Larvae were collected immediately after dissection (L3) or after 1, 2 or 3 days in culture, washed with Tris-EDTA buffer and then snap-frozen in liquid nitrogen. Ultrastructural examination by electron microscopy confirmed that these cultured larvae had started the moulting process, as the separation between the cuticle of L3 and the newly synthesized cuticle of L4 was evident in some of the cross-sections^52^. Nodular and skin microfilariae were purified as described previously^54,55^ and the adult worms were excised from collagenase-treated nodules as previously described^49^. Parasites were stored in trizol until RNA isolation.

### Optical mapping

Snap-frozen L3s were thawed and used to make agarose plugs using the CHEF Genomic DNA Plug Kit (Bio-Rad). Approximately 3 × 10^3^ L3s were spun at 1,000*g* for 5 min, resuspended in 32 μl cell suspension buffer and incubated in a 50 °C water bath; 53 μl of 2% clean-cut agarose, melted at 50 °C was added to the L3 and mixed gently before being transferred to a plug mould and stored at 4 °C. The plug was incubated in 100 μl proteinase K and 2.5 μl proteinase K reaction buffer at 50 °C for 1 day, before being washed five times, 1 h per wash, with gentle agitation, in 1× Wash buffer. The plug was stored at 4 °C until use. For optical mapping, DNA molecules were stretched and immobilized along microfluidic channels before digestion with the restriction endonuclease *Spe*I, yielding a set of restriction fragments ordered by their location in the genome. The fragments were fluorescently stained and visualized to determine the fragment sizes. Assembling overlapping fragment patterns of single-molecule restriction maps produced an optical map of the genome consisting of six large optical contigs with a total size of 92.54 Mb.

### Genome sequencing for assembly

Whole-genome sequence libraries (Supplementary Table 19) were generated from genomic DNA extracted from a single adult female *O. volvulus* worm and a single adult female *O. ochengi* worm, both washed thoroughly in 0.1 M EDTA. DNA was extracted using the Qiagen Genomic-tip kit. The ratio of host to parasite DNA was examined by qPCR using single-copy genes as markers. PCR-free 400–550 bp paired-end Illumina libraries were produced (one library per species) using a protocol based on a previously described method^56^ but using Agencourt AMPure XP beads for sample clean-up and size selection. Genomic DNA was precipitated onto beads after each enzymatic stage with an equal volume of 20% polyethylene glycol 6000 and 2.5 M sodium chloride solution. Beads were not separated from the sample throughout the process until after the adapter ligation stage. Fresh beads were then used for size selection. *O. volvulus g*enomic DNA was used to generate a 3 kb mate pair library using a modified SOLiD 5500 protocol adapted for Illumina sequencing^57^.

### Genome resequencing of sexed juvenile female and male worms

DNA was extracted from sexed juvenile male and female worms using the Promega Wizard kit, following the manufacturer’s instructions. Then, 400 bp fragment Illumina libraries were produced (Supplementary Table 19) following the protocol used for genome sequencing (see above), but using 12 cycles of amplification.

### Genome assemblies

The assembly of the genome was produced using a combination of sequencing, a *de novo* optical restriction map (Supplementary Fig. 1a) and extensive manual assembly improvement.

The initial assembly for *O. volvulus* was produced from a combination of short-fragment paired-end and mate-pair Illumina libraries. Short paired-end sequence reads were first corrected and initially assembled using SGA v0.9.7 (ref. 58). This draft assembly was then used to calculate the distribution of *k*-mers for all odd values of *k* between 41 and 81, using GenomeTools v.1.3.7 (ref. 59). The *k*-mer length for which the maximum number of unique *k*-mers were present in the SGA assembly was then used as the *k*-mer setting for de Bruijn graph construction in a second assembly with Velvet v1.2.03 (ref. 60). The mate-pair library was then used to further scaffold this Velvet assembly using SSPACE (ref. 61), followed by gap closing using Gapfiller (ref. 62) and IMAGE (ref. 63). A ‘bin’ assembly using the unaligned reads was incorporated into the main assembly and this assembly was scaffolded using SSPACE. The *O. ochengi* genome assembly was produced using the above method except that only a short-fragment paired-end (PCR-free) library was used and therefore the SSPACE scaffolding step was excluded. The *O. ochengi* assembly was found to be contaminated with sequences derived from the host (cow) genome. To identify contaminating contigs, PROmer (ref. 64) was used to compare the *Bos taurus* UMD 3.1 (ref. 65) assembly against the initial *O. ochengi* assembly and contigs with hits of greater than 97% identity covering more than 90% of the contig length were removed. This process removed ∼20 Mb of the *O. ochengi* assembly and this cleaned version was used for all comparisons reported here.

For *O. volvulus*, the output of the automated assembly process above produced an ‘interim’ assembly, which was improved by an extensive manual finishing effort, using GAP5 (ref. 66); scaffolds were extended, linked and, where possible, errors detected by REAPR (ref. 67) were fixed. The *de novo* optical map guided this process. The improved sequence scaffolds (totalling 90.9 Mb) were aligned against the optical contigs using MapSolver. Any miss-joins were resolved and new potential joins were investigated and executed where possible. Further automated gap closure was undertaken during this finishing process using IMAGE and Gapfiller, and the accuracy of the consensus sequence was improved using ICORN2 (ref. 68) with both short-fragment and mate-pair Illumina reads. Finally, REAPR was run to detect and break misassembled regions based on the mate-pair library data. The v.3 genome assembly resulting from this work was used for gene finding (Supplementary Table 2).

Although it was not possible to extend sequence data beyond the boundaries of the optical contigs, two additional joins could be made between four of the six superscaffolds, with evidence of these joins coming from read pairs joining the two ends and in one case sequence similarity between the two ends, and synteny with *C. elegans* and a *B. malayi* optical map (unpublished) supporting that, in each case, the two scaffolds being joined belong to the same chromosome. The v.4 genome assembly includes just these two additional joins, with minimum sizes for the introduced gaps (approximately 100 and 300 kb) estimated as the lengths of unjoined ‘overhangs’ in the optical mapping molecules. The final assembly thus has all four chromosome pairs represented by large contiguous scaffolds (Supplementary Table 2).

To assess the completeness of the assemblies, we ran CEGMA v2 (ref. 20), which reports the percentage of 248 highly conserved eukaryotic gene families that are present as full or partial genes in the assembly. For most eukaryotes, we would expect to see nearly 100% of CEGMA families represented by a full gene in the genome. Thus, CEGMA provides a measure of the completeness of the assembly for a species.

The *Wolbachia* endosymbiont genome sequence was assembled by first identifying scaffolds from the initial Velvet assembly that showed sequence similarity to published sequences of five published lambda phage clones containing *Wolbachia* sequence from Fenn and colleagues^69^ (a total of 70.8 kb). Nucmer^70^ identified a set of eight contigs totalling 113 kb that all had >99% similarity to the published clones over at least 500 bp and that all had very similar coverage (43–46×) in the Illumina data. These contigs also had more distant similarity to the published *Brugia malayi Wolbachia* sequence^71^. Applying a cutoff of depth between 42 and 47× coverage and PROMER similarity of at least 75% over 250 bp with the *B. malayi Wolbachia* sequence identified 44 contigs covering 926 kb that were putatively assigned to the *O. volvulus Wolbachia* sequence. Manual finishing using GAP5 then allowed us to order, extend and link these contigs to complete the *Wolbachia* genome assembly.

### Transcriptome sequencing and analyses

High-throughput transcriptome data were generated from the RNA of *O. volvulus* stage-specific parasites: nodular microfilariae, skin microfilariae, L2, L3, L3D1, L3D3, adult male and adult female worms. For all larval stages and adult worms, RNA was prepared using TRIzol and lysing matrix D (1.4 mm ceramic spheres) and a Fastprep24 (MP Biomedicals). RNAseq libraries were prepared following the RNAseq protocols of the Illumina mRNAseq Sample Prep kit and the Illumina TruSeq kit (Illumina). Transcriptome libraries were sequenced on Illumina HiSeq 2000 or MiSeq machines (Supplementary Table 19).

### Gene prediction

Gene predictions were conducted by various methods available in MAKER version 2.2.28 (ref. 72). The MAKER annotation pipeline consists of four general steps to generate high-quality annotations by taking into account evidence from multiple sources. First, assembled contigs are filtered against RepeatRunner^73^, RepBase^74^ and a species specific repeat library generated by Repeat Modeler (http://www.repeatmasker.org/RepeatModeler.html) using RepeatMasker (http://www.repeatmasker.org/) to identify and mask repetitive elements in the genome. Second, gene predictors Augustus 2.5.5 (ref. 75), GeneMark-ES 2.3a (self-trained)^76^ and SNAP 2013-02-16 (ref. 77) are employed to generate *ab initio* models that can use evidence within MAKER. Further species-specific gene models were provided to MAKER using comparative algorithms against the genome: genBlastG (ref. 78) output of *C. elegans* gene models (WormBase^79^) and RATT (Rapid Annotation Transfer Tool^80^) output of gene models from *B. malayi*, the taxonomically nearest reference genome. Third, a set of expressed sequence tags (ESTs), cDNAs and proteins from related organisms were aligned against the genome using BLASTN and BLASTX (ref. 81), respectively, and these alignments were further refined with respect to splice sites using Exonerate^82^. Finally, the EST and protein homology alignments, comparative gene models and the *ab initio* gene predictions were integrated and filtered by MAKER to produce a set of evidence informed gene annotations. The MAKER genome annotation pipeline was run three consecutive times. In the absence of a species-specific trained gene predictor, Augustus and SNAP were trained using CEGMA protein evidence gained from the default KOGs. The RNAseq data were mapped to the genome assembly using TopHat2 (ref. 83). The TopHat output includes the inferred positions of intron splice sites in the genomes and this information was fed into MAKER. The first run of MAKER was performed using the est2genome and protein2genome option with taxonomy-specific ESTs and cDNAs available from INSDC (ref. 84), including data from a large-scale EST project for *O. volvulus*^85,86^ and nematode protein sequences, respectively. Gene models obtained from the first run were used to train SNAP and models from the second run were used to train Augustus. With the trained models, MAKER was run a third time using a taxonomically broader protein set that included metazoan proteins from the UniProt Complete protein database^87^ and a subset of nematode proteomes from GeneDB (ref. 88). Finally, the MAKER gene set was filtered to remove less reliable gene models, as follows. First, MAKER gene models that were predicted based on Exonerate or BLASTX alignments and that did not overlap with any Augustus, genBlastG or RATT gene models were discarded, as they were probably due to spurious alignments. Second, MAKER gene models that encoded proteins of fewer than 30 amino acids were discarded. Third, if two different MAKER gene models overlapped in their coding sequence, the gene model with the worse score was discarded.

Because the *O. ochengi* genome assembly is much more fragmented than the *O. volvulus* assembly and there is no RNAseq evidence to guide gene finding, exons are frequently missed or complex gene models broken up in the annotation of this species.

### Identification of *Wolbachia* insertions in *O. volvulus* genome

The *O. volvulus Wolbachia* 956 kb genome contains 785 predicted protein-coding genes. Comparison of *w*Ov with the published *Wolbachia* of *O. ochengi* (*w*Oo)^89^ revealed the two genomes to be nearly identical (99.51%) but *w*Ov to be 1,937 bp larger, predominantly due to a small number of large indels. Approximately 4,400 single nucleotide polymorphisms (SNPs) were also identified, of which 57 variants may result in functional differences, including some that could be involved in the interaction of the symbionts with their hosts. The *O. volvulus* assembly was searched against the *w*Ov genome with NUCMER v3.06 using MAXMATCH, revealing a total of 531 matches. Of those, 486 were >100 bp and 7 were >1 kbp. Subsequently, the predicted *w*Ov proteins were used to search for regions with protein homology in the *O. volvulus* genome using TBLASTN as implemented in NCBI BLAST 2.2.21, with results reported in tabular format and an e-value threshold of 1e-15. The regions with matches in the *O. volvulus* assembly were extracted and searched against NT with BLASTN and NR with BLASTX as implemented in NCBI BLAST 2.2.21. Matches not meeting an e-value threshold of 1e-15 were discarded, as well as those without a best match to a bacterial gene/protein (which would include mitochondrial and nuclear mitochondrial sequences), yielding a list of putative nuwts in the *O. volvulus* genome (Supplementary Table 16). These regions were also searched with PRAZE (http://ber.sourceforge.net) against the predicted *w*Ov proteins to identify frameshifts, nonsense mutations and truncations.

### Comparative analysis of the predicted *O. volvulus* gene set

To help us examine the evolution of gene families and to identify the families of genes lost or expanded in *O. volvulus*, we ran the Ensembl Compara pipeline^22^. Ensembl Compara is a pipeline that clusters genes into families based on all-against-all blast scores, produces multiple alignments and phylogenies for each gene family, and predicts paralogy and orthology relationships by reconciling reconstructed gene trees with the phylogeny for the taxa included. Along with *O. volvulus* and *O. ochengi*, we included published filarial nematode genomes (*Dirofilaria immitis*, *Wuchereria bancrofti*, *B. malayi* and *Loa loa*), *Ascaris suum* as the only other clade III nematode species for which genome data has been published, *C. elegans* as the model free-living nematode species and the clade I parasitic nematode *Trichuris muris* as an outgroup to all of these. The phylogenetic tree of these species used as input to Compara was constructed from existing evidence on relationships between filarial species and was identical in topology to that shown in Fig. 2. Using the Compara Perl API to query our custom Ensembl Compara database, we identified gene families with multiple expansions or losses in *O. volvulus* relative to the other comparator species. We removed all the ‘dubious’ duplications (those with a duplication confidence score ≤0) and defined a gene family as expanded if there were at least two gene duplication events reported in *O. volvulus*. To help us in identifying gene families that are expanded in *O. volvulus*, we looked at both the gene count and the total protein length per species in these families, as the latter statistic is less sensitive to fragmented gene models.

A phylogeny of the nine nematode species was generated from 3,148 single-copy Compara gene families that have genes from at least seven species. Sequences for each gene family were aligned using Mafft v7.205 (ref. 90) in automatic (−auto) mode; these alignments were then trimmed with GBlocks v0.91b (ref. 91) with default parameters, and trimmed alignments were then concatenated to produce a single global data matrix. The phylogeny was then inferred using RAxML v8.0.24 (ref. 92), with each gene family alignment treated as a separate partition using the best-fitting model for that alignment (minimum corrected Akaike information criterion (AIC)) from the empirical amino acid substitution models available in that version of RAxML. The preferred phylogeny was estimated with 10 random addition-sequence replicates and support for splits on the tree estimated using 100 bootstrap replicates.

### Functional annotation of genes and gene families

Gene ontology (GO) terms were assigned to genes by transferring GO terms from *C. elegans* orthologues based on the Ensembl Compara approach for transferring GO terms to orthologues in vertebrate species^22^, but modified for improved accuracy in transferring GO terms across phyla. Manually curated GO annotations were downloaded from the GO Consortium website^93^ and, for a particular predicted protein in the present study, the manually curated GO terms were obtained for its *C. elegans* orthologues and then transferred to our predicted protein. GO terms of the three possible types (molecular function, cellular component and biological process) were assigned to predicted proteins in this way. Additional GO terms were identified using InterproScan^94^. We developed two pipelines to annotate the Compara gene families: one for the assignment of GO terms to genes and another to assign product description to the gene families.

### Chemogenomics screening

A computational target-based approach was used to screen FDA^95^-approved drugs across all World Health Organization Anatomical Therapeutic Classes (WHO ATC)^96^ to identify drugs with potential for use as anthelmintics. For protein target identification, we built a Compara database containing all the proteins in release WBPS1 of WormBase ParaSite together with proteins from the 2010-07 Ensembl gene build for the human reference genome assembly GRCh37. An initial broad stroke approach identified as a potential drug target any *O. volvulus* protein belonging to the same gene family in this database as a human protein annotated as a drug target in ChEMBL. A more stringent approach identified *O. volvulus* proteins as targets only if they were one-to-one orthologues of a human target protein in the same Compara database. Further filtering of the identified proteins was based on molecular weight, number of transmembrane domains, number of disulfide bonds and life-cycle stage. We identified approved drugs with annotated targets in ChEMBL for targets with *O. volvulus* homologues identified by both the stringent one–one orthologue ‘individual’ protein approach and the more comprehensive approach using Compara families. Both drug sets were filtered on mode of administration (that is, oral, topical and parenteral). Drugs administered solely via an intravenous route were removed from the set. Further filtering was carried out to restrict the drug set to those with a single protein target, thus excluding drugs targeting protein complexes. We created a drug–protein family interaction network to represent family-level drug identification using the network visualization tool Cytoscape^97^. The edges (lines) connect nodes (circles) representing drugs and their protein family targets, respectively (Supplementary Fig. 8). This allowed protein families to be identified as having at least one drug target and those with particularly high numbers to be highlighted as potentially the most ‘druggable’ targets.

### Ion channels and other anthelmintic drug targets

Nucleotide sequences for selected ion-channel genes were aligned as codons using MAFFT (ref. 98) and regions with uncertainty caused by high levels of sequence divergence and differing sequence length were removed. Maximum-likelihood phylogenies were inferred using PhyML (v20120412)^99^ with branch node significance determined from the Shimodaira–Hasegawa (SH) statistics and from 100 bootstrap replicates.

### Metabolic reconstruction and FBA

For each organism, an initial set of EC predictions was obtained from several methods: (1) DETECT v2.0 (cutoff ILS ≥0.9, ≥5 positive hits^100)^, (2) BLASTP^81^ (e-value 1e-10 against SWISSPROT^101^ enzymes), (3) PRIAM^102^ enzyme rel. Feb-2014 (minimum probability >0.5, profile coverage >70%, check catalytic - TRUE), (4) KAAS^103^, (5) EFICAz^104^ and (6) EC assignments from BRENDA^105^. From these assignments, a set of high-confidence predictions was derived as follows from BRENDA, DETECT and reactions identified by both PRIAM and KAAS. Reaction assignments to metabolic pathways and pathway hole filling were performed using Pathway Tools v18.0 (ref. 106). Those predictions with support from either EFICAz, PRIAM or BLASTP were used to augment the high-confidence set of predictions. In addition, novel Pathway Tool predictions to genes that were previously unannotated were also incorporated.

From our draft networks, we completed our metabolic reconstruction, starting with our high-confidence sets of enzymes for both *O. volvulus* and *L. loa*. In the case of *O. volvulus*, we also added 100 enzymes (ECs) unique to its *Wolbachia* endosymbiont. We used KEGG as a guide to find the reactions catalysed by individual enzymes. Reactions that use macromolecules to derive certain metabolites, specifically those reactions that contain the same compound (such as DNA) on both sides of the equation, were considered uninformative and filtered. We further modified some reactions from their original KEGG formulation as they contained a glycan identified in KEGG as equivalent to a compound also present in the reconstruction. Reaction directionalities were defined with reference to KEGG, with reactions marked as either reversible (lower bound −1,000 and upper bound 1,000 mmol (g_DW_ h)^−1^) or irreversible (with lower bound 0 and upper bound 1,000 mmol (g_DW_ h)^−1^ or lower bound −1,000 and upper bound 0 mmol (g_DW_ h)^−1^ depending on directionality). We added a non-growth-associated maintenance (NGAM) equation, accounting for the organism’s needs outside of growth, to be at least 5 mmol (g_DW_ h)^−1^. We allowed for glucose uptake at a maximum of 10 mmol (g_DW_ h)^−1^ and initially allowed for diffusion of water, oxygen, carbon dioxide, ammonia, diphosphate, phosphate and ethanol. We also allowed, by default, the transport of all amino acids into the system. Model biomass was defined with reference to a previous metabolic reconstruction for the parasites *Toxoplasma gondii*^107^ and *Leishmania major*^108^ (Supplementary Table 13).

Pathway gap-filling for both *O. volvulus* and *L. loa* was first performed to ensure production of biomass components and subsequently to complete metabolic pathways missing a limited number of reactions. In this process, we identified from the literature additional metabolites as possibly important for growth and added these as part of the biomass (biomass components 46–49, Supplementary Table 13)^109–111^. We also added as part of the biomass such important cofactors as NADH, NADP+ and FAD (biomass components 50–52). In all, we added 68 KEGG reactions for *O. volvulus* and 50 for *L. loa*. In addition, two reactions previously added for gap-filling purposes for *O. volvulus* were found to have some evidence through reciprocal BLAST against *L. loa* (R04230 and R04231 corresponding to EC 6.3.2.5). To enable arachidonic acid metabolism from linoleic acid, two reactions (common to both reconstructions and with gene evidence: R03814_1 (1.14.19.3) and R00390_1 (6.2.1.3)) were modified from their KEGG formulations, while one reaction (common to both reconstructions: N00001) was custom-made. Further modifications involve changing the directionalities of R01665 (2.7.4.14) and R01664 (3.1.3.5) to ensure CMP was not a dead end; R00086 (ATP phosphohydrolase; linked to many ECs) was modified to allow proton export. For both models, we ensured that the same utility reactions were added so that the models were comparable. Thus, in all, each reconstruction consists of 63 utility reactions, namely 43 transport reactions, 8 sinks, 4 demand reactions and 8 diffusion reactions (a diffusion reaction was added when allowing for the movement of bicarbonate). Moreover, both reconstructions contain six irreversible reactions to ensure that specific metabolites (hexanoyl-CoA, octanoyl-CoA, decanoyl-CoA, lauroyl-CoA, tetradecanoyl-CoA and palmitoyl-CoA, with KEGG IDs C05270, C01944, C05274, C01832, C02593 and C00154, respectively) are considered as contenders for the more general compound acyl-CoA (KEGG ID: C00040) involved in such pathways as glycerophospholipid metabolism. Note that we predict *L. loa* to be a threonine autotroph through pathway gap filling. In particular, the presence of the enzyme EC 1.1.1.103 (present in *L. loa* only) specializing in glycine, serine and threonine metabolism suggests the possible production of threonine from glycine. Consequently, the reaction catalysed by EC 2.3.1.29 (R00371) was added to complete the circuit.

Shared between *O. volvulus* and *L. loa* are 628 reactions and 250 distinct EC numbers. Most of the additional reactions present in the *O. volvulus* reconstruction (139 reactions) are contributed by *w*Ov (100 reactions), none of which are encoded in the *L. loa* genome.

FBA^34,35^ was used to simulate growth of the worms. A naive objective function was set as maximizing yield (biomass production) for both nematodes. The COBRA Toolbox (version 1.3.4) was used in conjunction with MATLAB to perform simulations^112^. Single-reaction knockout experiments were performed by blocking the flow through individual reactions (that is, setting the upper and lower bounds as zero) and then measuring the yield through the biomass function. Growth ratios were found by dividing the yield following the block by the yield in an unrestrained model (that is, with the reaction unblocked). Reactions were defined as essential if their growth ratio was predicted to be less than 0.1 upon removal of that reaction. Further details of the metabolic reconstruction are provided in Supplementary Table 13.

In these analyses no attempt was made to partition reactions due to the challenge of accurately assigning reactions to distinct intracellular compartments (for example, mitochondria, golgi and *wOv*), as well as determining the specific small-molecule transporter activities that allow metabolites to move between compartments. Instead, metabolites were assumed to be freely available across reactions, an assumption, as previously mentioned, that is expected to minimize false-positive essential reaction predictions at the expense of increasing the likelihood false-negative predictions.

## Supplementary Material

Data

INFO

tables

## Figures and Tables

**Table 1:** Overview of the metabolic pathways included in the metabolic reconstructions.

		Number of reactions in pathway			Number of reactions predicted essential*		
Superpathway	Metabolic pathway	*L. loa*	Ov and *w*O*v*	*w*O*v* only	*L. loa*	Ov and *w*O*v*	*w*O*v* only
Amino acid metabolism	Alanine, aspartate and glutamate metabolism	14	15	3	1	1	0
	Arginine and proline metabolism	26	25	2	3	3	0
	Biosynthesis of amino acids	37	41	9	3	3	0
	Cysteine and methionine metabolism	17	19	2	4	4	0
	Glycine, serine and threonine metabolism	17	17	3	1	1	0
Biosynthesis of other secondary metabolites	Streptomycin biosynthesis	4	4	0	2	2	0
Carbohydrate metabolism	Amino sugar and nucleotide sugar metabolism	23	23	3	4	4	0
	Butanoate metabolism	8	8	0	1	0	0
	Inositol phosphate metabolism	12	12	0	3	3	0
	Pentose phosphate pathway	17	18	0	2	2	0
	Propanoate metabolism	14	14	1	1	1	0
	Pyruvate metabolism	20	20	1	1	1	0
Energy metabolism	Methane metabolism	16	16	0	2	2	0
Lipid metabolism	Arachidonic acid metabolism	16	14	0	3	3	0
	Biosynthesis of unsaturated fatty acids	14	15	2	7	2	0
	Fatty acid biosynthesis	3	15	32	1	1	0
	Fatty acid degradation	32	32	0	19	0	0
	Fatty acid elongation	28	28	0	24	0	0
	Fatty acid metabolism	48	57	32	31	2	0
	Glycerolipid metabolism	9	9	0	2	2	0
	Glycerophospholipid metabolism	22	22	1	6	6	0
	Sphingolipid metabolism	16	16	0	5	5	0
Metabolism of cofactors and vitamins	Nicotinate and nicotinamide metabolism†	11	12	1	1	0	1
	One carbon pool by folate	10	10	6	1	0	0
	Pantothenate and CoA biosynthesis	11	11	1	3	3	0
Metabolism of other amino acids	Beta-alanine metabolism	9	9	0	1	1	0
	Glutathione metabolism	19	18	0	2	2	0
Metabolism of terpenoids and polyketides	Terpenoid backbone biosynthesis	12	12	2	8	8	0
	Tetracycline biosynthesis	1	1	0	1	1	0
Nucleotide metabolism	Purine metabolism	53	63	9	7	3	0
	Pyrimidine metabolism	47	51	7	7	5	0
Transport reactions	Extracellular transport	43	43	0	23	18‡	0
	Total (non-redundant) number of reactions in pathways	428	455	72	86	49	1
	Total number of reactions in reconstructions	777	796	100	112	70	1

* One reaction was essential either from *L. loa* (with or without genetic evidence); *O. volvulus* (Ov) and *Wolbachia* (*w*O*v*) with reactions in the *O. volvulus* reconstruction (both from gene annotation and added in pathway gap-filling) not being contributed by *w*O*v* only; or *w*O*v* with reactions in the Ov reconstruction supported by genetic evidence only from *w*O*v*.

† Only a single reaction, in the nicotinate and nicotinamide pathway, is essential and uniquely provided by *w*O*v*. Transport reactions for both nematode and *Wolbachia* lack genetic evidence therefore making *w*O*v* only and *O. volvulus* and *w*O*v* transporters indistinguishable.

‡ Note that of the 18 transport reactions essential to *O. volvulus*, all are essential to *L. loa* as well except for threonine transport. Also reported is the total number of reactions belonging to the pathways listed here, as well as the total number of reactions in the reconstructions.

**Table 2:** The top 16 *O. volvulus* targets and their predicted drugs.

Ov gene ID	Function of family	Drugs (WHO name)	ATC level 3 classification
OVOC2713	Ribonucleotide reductase, small chain	Fludarabine phosphate, clofarabine, gemcitabine, (hydroxyurea), gallium nitrate	Antimetabolites, other antineoplastic agents.
OVOC11146	Calcineurin-like phosphoesterase	Tiagabine	Antiepileptics
OVOC215	Sodium neurotransmitter symporter family	Imipramine, clomipramine, amitriptyline, nortriptyline, protriptyline, amoxapine, fluoxetine, citalopram, paroxetine, sertraline, fluvoxamine, escitalopram, trazodone, nefazodone, venlafaxine, milnacipran, duloxetine, desvenlafaxine, vilazodone, vortioxetine, (levomilnacipran, chlorphentermine)	Antidepressants (unclassified)
OVOC3747	Ion transport protein	Dronedarone, (nimodipine, felodipine)	Antiarrhythmics, class I and III (selective calcium channel blockers with mainly vascular effects)
OVOC1986	KQT-1 potassium channel	Dronedarone	Antiarrhythmics, class I and III
OVOC1111	Receptor family ligand binding region	Baclofen, oxybate	Muscle relaxants, central acting agents, null
OVOC5191	G-protein coupled GABA receptor activity		
OVOC12632 OVOC12637 OVOC12635 OVOC968	NADH-ubiquinone oxidoreductase chains 1, 4 and 5; probable NADH dehydrogenase [ubiquinone] iron-sulfur protein 7	Metformin	Blood glucose lowering drugs excluding insulin
OVOC6309	3-oxo-5-α-steroid 4-dehydrogenase	Dutasteride	Drugs used in benign prostatic hypertrophy
OVOC10592	ERG2 and sigma1 receptor like protein	Pentazocine, (dextromethorphan)	Opiods, cough suppressants (other nervous system drugs)
OVOC8585	C-terminal tandem repeated domain in type 4 procollagen	Xiaflex, (ocriplasmin)	Other drugs for disorders of the musculoskeletal system (other ophthalmologicals)
OVOC10695	Glycosyl transferase family 21	Miglustat, eliglustat	Other alimentary tract and metabolism products
OVOC4110	Amidase	Acetaminophen	Other analgesics and antipyretics

The table shows anatomical therapeutic classification groups for drugs identified as potential repurposing candidates. For drugs in parentheses, the mechanism is shown in similar style parentheses in the ‘ATC level 3 classification’ column.
