# Supplementary material for: The genome of Onchocerca volvulus, agent of river blindness
Source: Nat Microbiol. Author manuscript; Available in PMC 2017 Feb 15. (PMC5310847; doi:10.1038/nmicrobiol.2016.216)
Supplement: INFO [file NIHMS848855-supplement-INFO.pdf]

The genome of *Onchocerca volvulus*, agent of river blindness

Cotton *et al.*

Supplementary Information:

Supplementary Figure 1. Optical maps.

Supplementary Figure 2. Patterns of read coverage and heterozygosity in different sequencing libraries.

Supplementary Figure 3. Functional classification of the putative proteome and the unique proteins of *O. volvulus.*

Supplementary Figure 4. Synteny and colinearity of *O. volvulus* and *O. ochengi* genomes.

Supplementary Figure 5. Alignment of conserved regions of collagen prolyl hydroxylase proteins.

Supplementary Figure 6. *O. volvulus* Nuclear Hormone Receptors.

Supplementary Figure 7. Nucleotide sequences for pLGIC subunits from the glutamate- and GABA- gated chloride-channel family.

Supplementary Figure 8. *O. volvulus* protein families identified as potential targets of FDA-approved drugs.

Supplementary Table 2. Properties of assemblies and annotations for *Onchocerca volvulus*, *O. ochengi* and other filarial nematodes.

Supplementary Table 3. Assignment of scaffolds to autosomes and sex chromosomes based on coverage in male and female sequencing libraries.

Supplementary Table 4. Conserved eukaryotic genes from CEGMA set missing from *O. volvulus* and other filarial nematodes.

Supplementary Table 5. Repeat content of the *Onchocerca volvulus* genome.

Supplementary Table 6. BLAST hits and sequence similarity between *O. volvulus* genes and genes in other nematodes

Supplementary Table 7. Putative *O. volvulus* Y-specific contigs and the genes present on these contigs, including gene product names inferred from homology of PFAM domain annotations.

Supplementary Table 8. Number of gene copies per species in protein families expanded in *O. volvulus*.

Supplementary Table 9. List of classical and orphan nuclear receptors identified in the *O. volvulus* genome and homologues in other species.

Supplementary Table 10. Genes participating in embryological and developmental functions in *C. elegans* and their homologues in *O. volvulus*.

Supplementary Tables 11. Set of reactions and metabolites used in the metabolic reconstruction of *O. volvulus*.

Supplementary Tables 12. Set of reactions and metabolites used in the metabolic reconstruction of *Loa loa*.

Supplementary Tables 13. Details of metabolites and their relative contributions to the biomass function for *O. volvulus* and *L. loa*, and details of glycan IDs that were replaced by compound IDs in the metabolic reconstructions.

Supplementary Table 14. List of FDA approved drugs that could potentially target 51 individual *O. volvulus* proteins.

Supplementary Table 15. Analysis of coding sequence variants and putative functional differences between *w*Ov and *w*Oo identified using SNPeff.

Supplementary Table 16. Gene-based identification of Nuwts in the *O. volvulus* genome.

Supplementary Table 17. Nuwts detected by nucmer that are >1 kb in the large scaffolds.

Supplementary Table 18. *O. volvulus* proteins of the innate immune system.

Supplementary Table 19. Details of DNA and RNA sequencing libraries and accession numbers.

Supplementary Data: Excel tables of Ensembl Compara data providing information on gene families and enriched GO terms that are specific to *Onchocerca volvulus*, and to different lineage levels: *Onchocercidae*, *Filarioidea,* and *Chromadorea* (includes *O. ochengi*, *Dirofilaria immitis*, *Wuchereria bancrofti*, *Brugia malayi*, *Loa loa* and *Ascaris suum*).

Supp_Fig.1

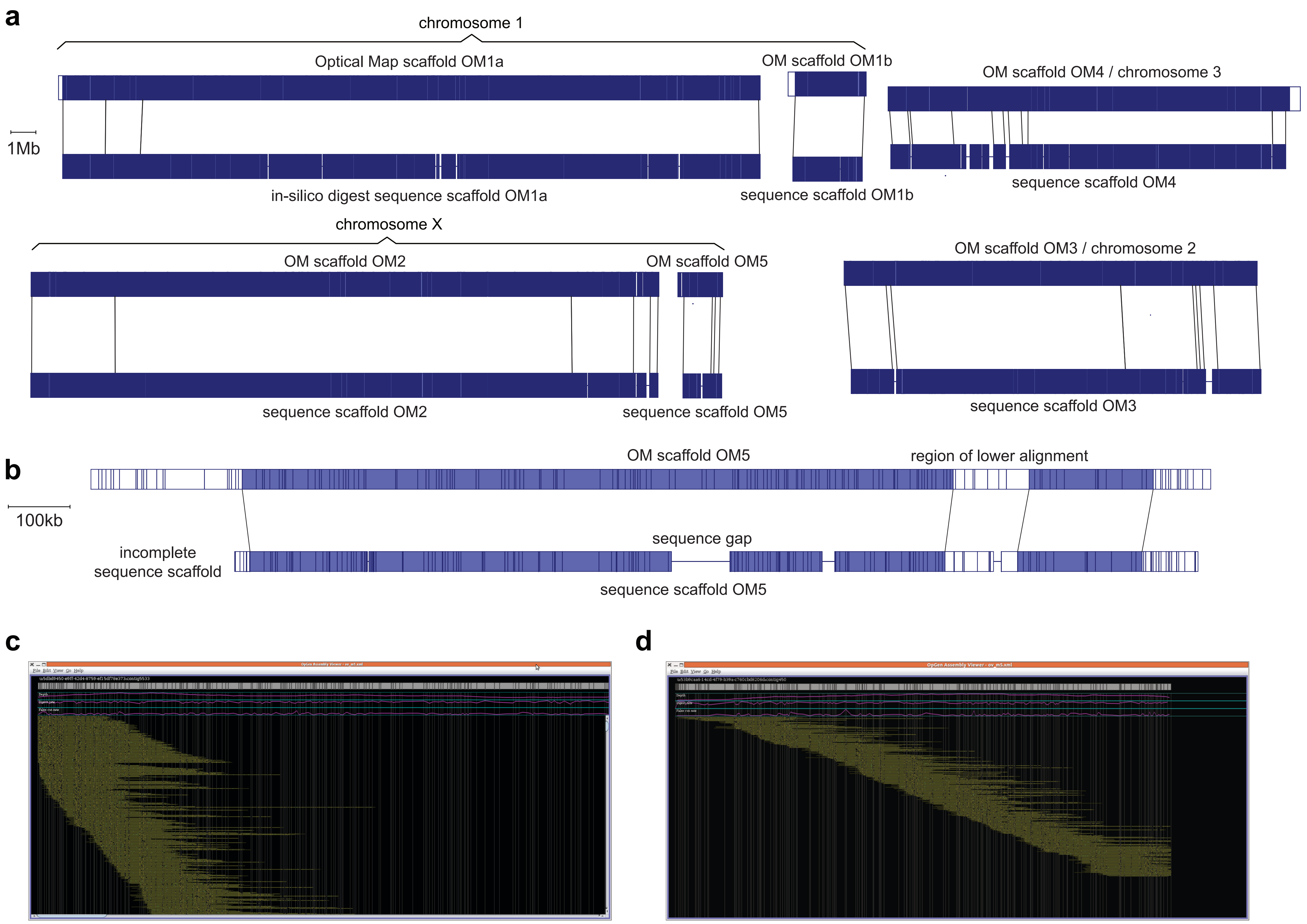
a
chromosome 1
Optical Map scaffold OM1a
OM scaffold OM1b
OM scaffold OM4 / chromosome 3
1Mb
in-silico digest sequence scaffold OM1a
sequence scaffold OM1b
sequence scaffold OM4
chromosome X
OM scaffold OM2
OM scaffold OM5
OM scaffold OM3 / chromosome 2
sequence scaffold OM2
sequence scaffold OM5
sequence scaffold OM3
b
OM scaffold OM5
region of lower alignment
100kb
sequence gap
incomplete
sequence scaffold
sequence scaffold OM5
c
d

Supplementary Figure 1. Optical maps. (**a**) Genome-wide optical map for *Onchocerca volvulus*, allowing contiguation and scaffolding of sequence data into 6 large scaffolds. Optical map (above) and in-silico digested sequence scaffolds (below) are shown for each chromosome. Individual restriction fragments are not visible at this scale, but the largest gaps in the sequence assembly and small areas in the optical map not covered by large sequence scaffolds are shown. Vertical lines delineate areas of alignment between the two maps. (**b**) Detailed view of the smallest optical map scaffold (OM5), showing individual restriction fragments (cut sites are shown as vertical lines within rectangles and showing gaps, uncovered regions and missing sequence regions. (**c**) and (**d**) show individual digested molecules aligned against optical map scaffolds. (**c**) shows a likely chromosome end, where all molecules finish at the end of the map to give a 'blunt' appearance. (**d**) shows an optical map scaffold end that is not a chromosome end, as molecule alignments 'tail off' at the end of the map.

Supp_Fig.2

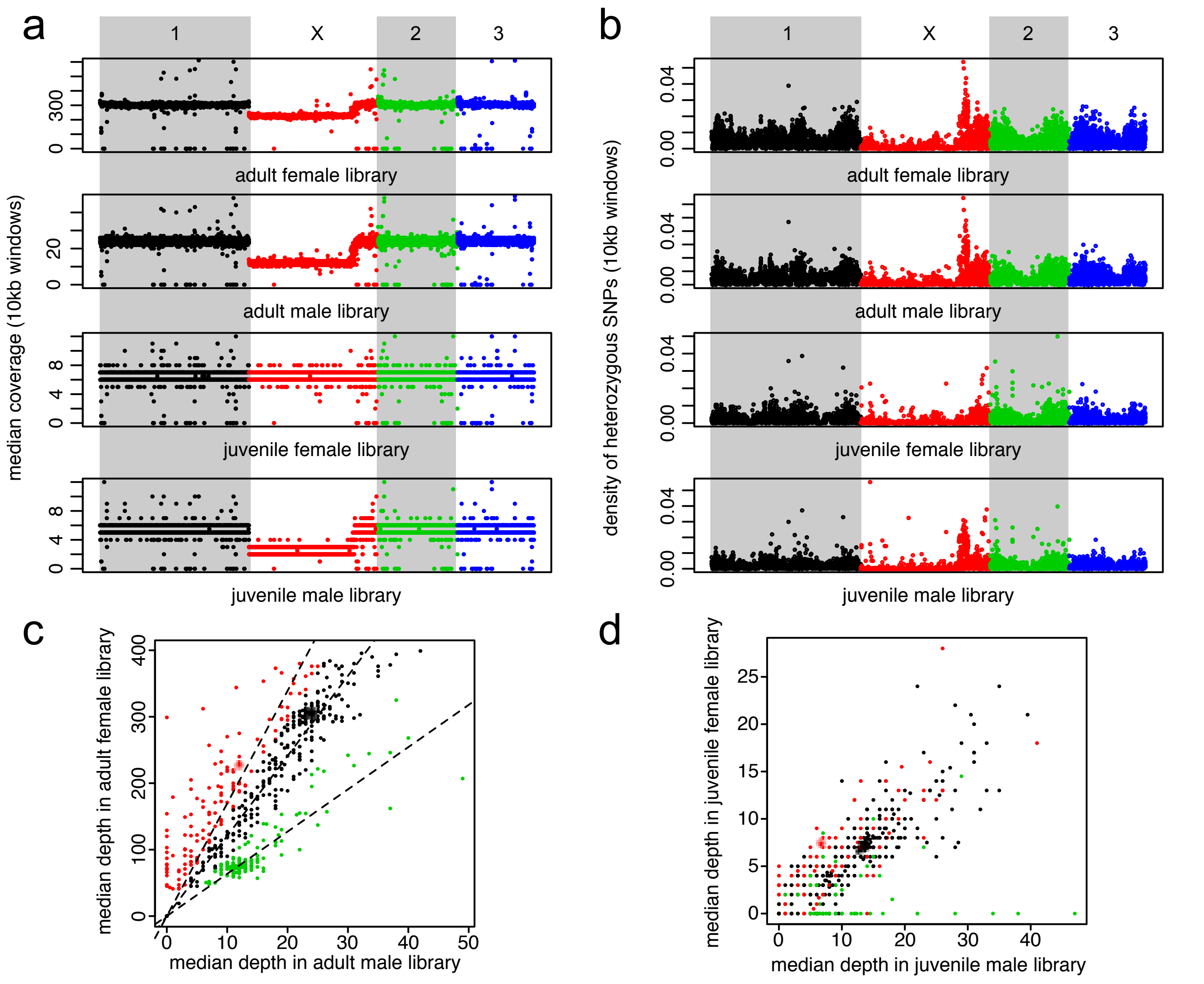
a
1
X
2
3
median coverage (10kb windows)
300
0
adult female library
20
0
adult male library
8
4
0
juvenile female library
8
4
0
juvenile male library
b
1
X
2
3
density of heterozygous SNPs (10kb windows)
0.04
0.00
adult female library
0.04
0.00
adult male library
0.04
0.00
juvenile female library
0.04
0.00
juvenile male library
c
median depth in adult female library
400
300
200
100
0
0
10
20
30
40
50
median depth in adult male library
d
median depth in juvenile female library
25
20
15
10
5
0
0
10
20
30
40
median depth in juvenile male library

Supplementary Figure 2. Patterns of read coverage and heterozygosity in different sequencing libraries. (a) Median depth of sequencing read coverage for non-overlapping 10kb windows across chromosomes. Different colored points represent windows from different scaffolds. We see consistently lower coverage across most of chromosome 2 in male samples and in adult females, which are likely gravid with both male and female offspring, but not in (virgin) juvenile females. The drop in coverage coincides with reduced heterozygosity across the 'low coverage' part of chromosome 2, as expected for sex chromosomes. There is also a 'spike' in heterozygosity specifically at the boundary between low and high coverage parts of this scaffold. **(b)** Proportion of heterozygote genotypes in 10 kb windows along the large scaffolds. **(c)** Coverage for all contigs and scaffolds in the assembly for adult samples. **(d)** Coverage for all contigs and scaffolds in the assembly for juvenile female and male worms. Colors of dots distinguish scaffolds assigned to different categories based on the ratio of coverage between adult male and female samples for autosomes (black), X-chromosome specific regions (red) and Y-chromosome specific regions (green) assuming an XY-genotype in male worms and that the 'adult female' sample contains a 50:50 proportion of male and female genotypes. Larger translucent points represent the chromosomes, with chromosome X split into the two regions of different coverage, with the low-coverage region colored red. (c) Shows the same scaffolds colored as in (b). While chromosomes show consistent coverage patterns expected from (c), most putative X-specific (red) small scaffolds do not, and only a subset of Y-specific scaffolds (green) show the expected zero coverage in the juvenile female sample. We propose that many of the small scaffolds instead represent variant haplotypes present in some samples but absent in others.

Supp_Fig. 3

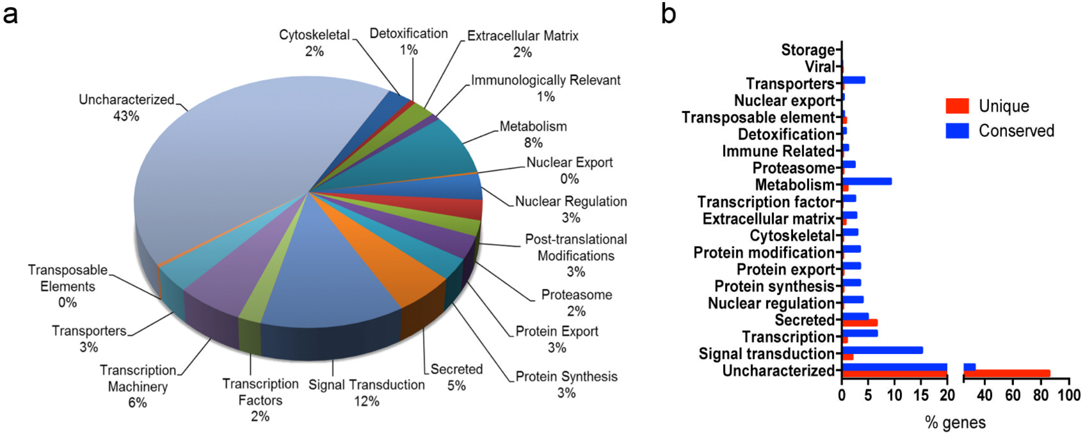
a
Cytoskeletal 2%
Detoxification 1%
Extracellular Matrix 2%
Immunologically Relevant 1%
Metabolism 8%
Nuclear Export 0%
Nuclear Regulation 3%
Post-translational Modifications 3%
Proteasome 2%
Protein Export 3%
Protein Synthesis 3%
Secreted 5%
Signal Transduction 12%
Transcription Factors 2%
Transcription Machinery 6%
Transporters 3%
Transposable Elements 0%
Uncharacterized 43%
b
Storage
Viral
Transporters
Nuclear export
Transposable element
Detoxification
Immune Related
Proteasome
Metabolism
Transcription factor
Extracellular matrix
Cytoskeletal
Protein modification
Protein export
Protein synthesis
Nuclear regulation
Secreted
Transcription
Signal transduction
Uncharacterized
Unique
Conserved
0 5 10 15 20 40 60 80 100
% genes

Supplementary Figure 3. Functional classification of the putative proteome and the unique proteins of *O. volvulus.* (a) Pie chart showing the functional distribution of the 12,143 putative proteins in *O. volvulus* and (b) the functional distribution of *O. volvulus* unique (<30% max score, blue bars) and conserved subsets (>30% max score, with other nematodes, red bars) of proteins.

Supp_Fig.4

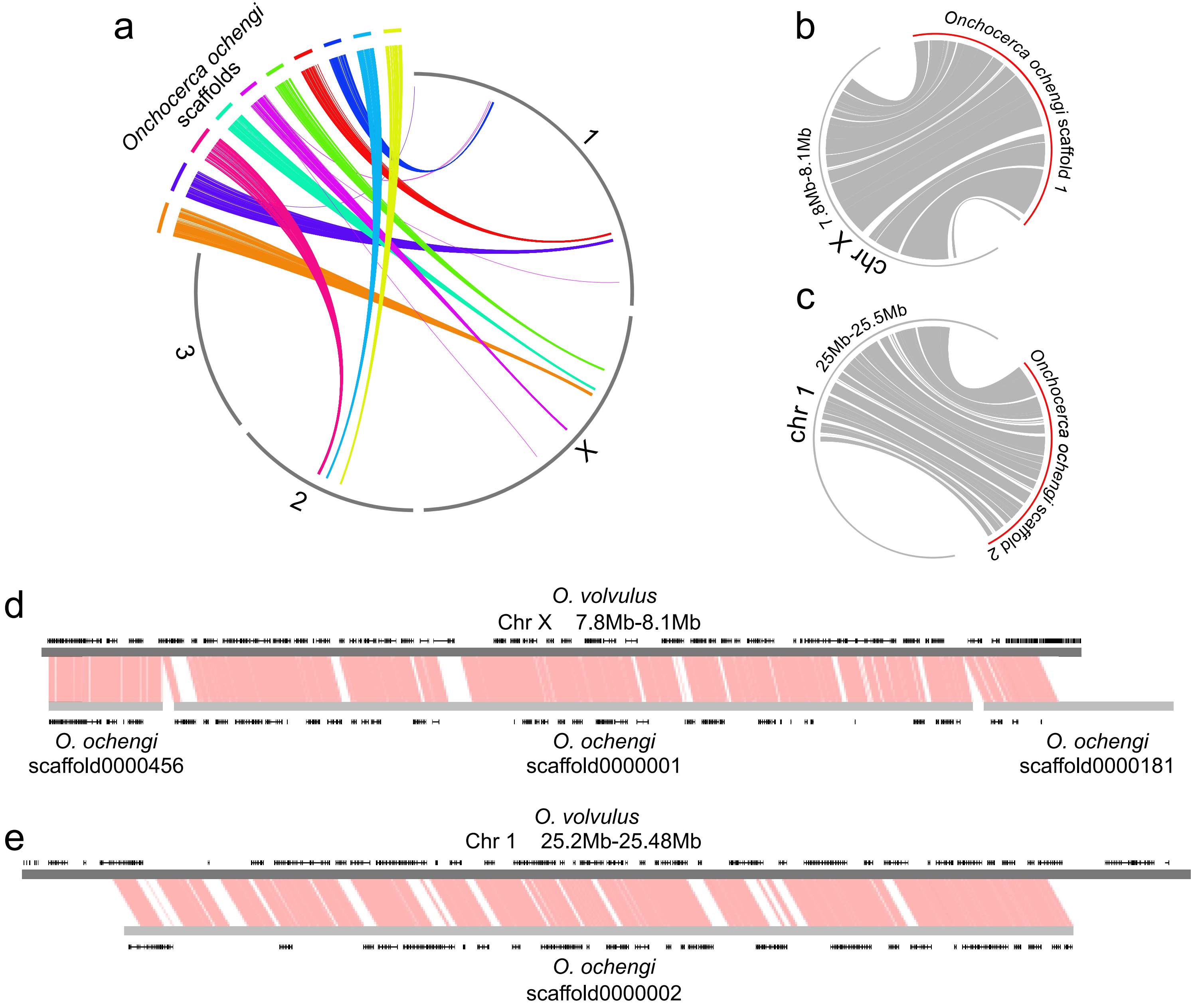

a
Onchocerca ochengi scaffolds
1
2
3
X
b
Onchocerca ochengi scaffold 1
chr X 7.8Mb-8.1Mb
c
25Mb-25.5Mb
chr 1
Onchocerca ochengi scaffold 2
d
O. volvulus
Chr X 7.8Mb-8.1Mb
O. ochengi
scaffold0000456
O. ochengi
scaffold0000001
O. ochengi
scaffold0000181
e
O. volvulus
Chr 1 25.2Mb-25.48Mb
O. ochengi
scaffold0000002

Supplementary Figure 4. Synteny and colinearity of *O. volvulus* and *O. ochengi* genomes. (a) Comparison of the ten largest *O. ochengi* scaffolds against *O. volvulus* genome assembly. Links show all PROmer hits of at least 98% similarity, colored by *O. ochengi* source. For clarity, *O. ochengi* scaffolds are shown 10x larger than *O. volvulus* scaffolds. *O. ochengi* scaffolds all map to unique regions of *O. volvulus*, confirming synteny between the two genomes. **(b-e)** Detailed views of the same comparisons for the two largest *O. ochengi* scaffolds with relevant regions of *O. volvulus* scaffolds, confirming colinearity of these regions between the two genomes. **(d)** and **(e)** also show annotated gene models (black rectangles show exons, with horizontal lines connecting exons within a gene), showing that most genes are in the same locations and have the same structure, but that more complex multi-exon gene structures are missed in *O. ochengi* annotation, due to the lack of RNAseq evidence needed to annotate these genes correctly. Panel (**d**) shows *O. ochengi* scaffolds with similarity to adjacent regions of *O. volvulus*, indicating that the colinearity is not restricted to the largest *O. ochengi* scaffolds. Both genomes are shown to the same scale in panels (**b-e**). For clarity, overlapping hits in different reading frames are removed for panels (**d**) and (**e**).

Supp_Fig.5

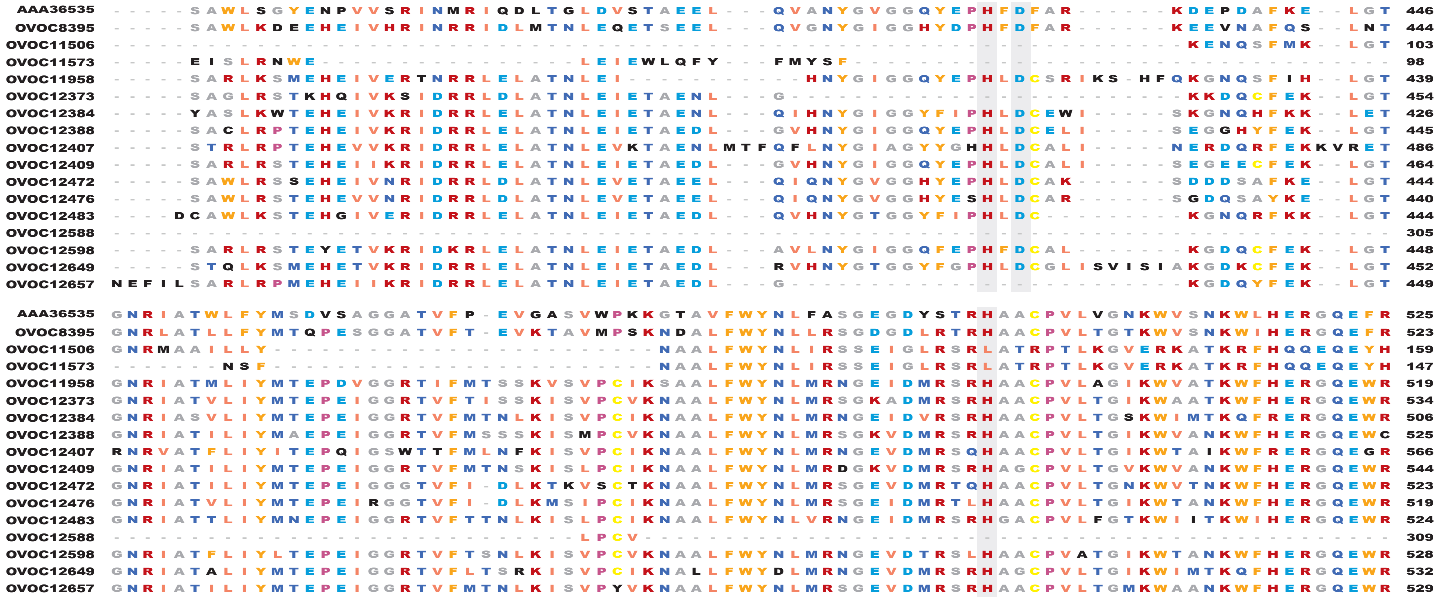
AAA36535 446
OVOC8395 444
OVOC11506 103
OVOC11573 98
OVOC11958 439
OVOC12373 454
OVOC12384 426
OVOC12388 445
OVOC12407 486
OVOC12409 464
OVOC12472 444
OVOC12476 440
OVOC12483 444
OVOC12588 305
OVOC12598 448
OVOC12649 452
OVOC12657 449
AAA36535 525
OVOC8395 523
OVOC11506 159
OVOC11573 147
OVOC11958 519
OVOC12373 534
OVOC12384 506
OVOC12388 525
OVOC12407 566
OVOC12409 544
OVOC12472 523
OVOC12476 519
OVOC12483 524
OVOC12588 309
OVOC12598 528
OVOC12649 532
OVOC12657 529

Supplementary Figure 5. Alignment of conserved regions of collagen prolyl hydroxylase proteins. The alignment shows part of a multiple alignment of all *O. volvulus* collagen prolyl hydroxylase proteins annotated together with a human orthologue (AAA36535). The histidine and aspartic acid residues (shaded) that are key to the active site of the collagen prolyl hydroxylase enzyme are conserved in *O. volvulus* genes. A previously published *O. volvulus* sequence (AAL08488) was almost identical to gene model OVOC12598, with identical predicted protein lengths and differing at just 5 amino acid positions out of 571.

Supp_Fig. 6

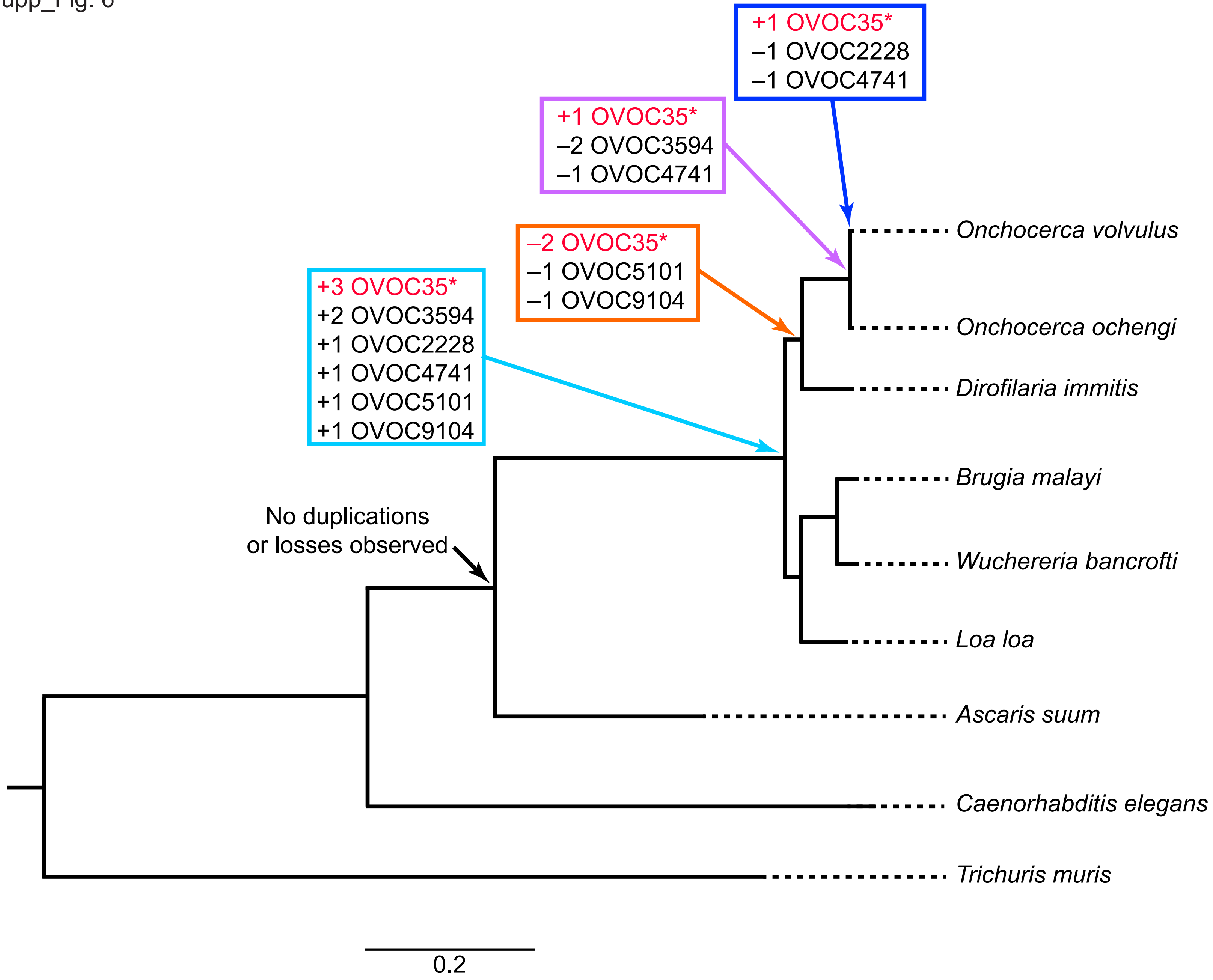

+1 OVOC35*
–1 OVOC2228
–1 OVOC4741
+1 OVOC35*
–2 OVOC3594
–1 OVOC4741
–2 OVOC35*
–1 OVOC5101
–1 OVOC9104
+3 OVOC35*
+2 OVOC3594
+1 OVOC2228
+1 OVOC4741
+1 OVOC5101
+1 OVOC9104
No duplications or losses observed
Onchocerca volvulus
Onchocerca ochengi
Dirofilaria immitis
Brugia malayi
Wuchereria bancrofti
Loa loa
Ascaris suum
Caenorhabditis elegans
Trichuris muris
0.2

Supplementary Figure 6. *O. volvulus* Nuclear Hormone Receptors. Ensembl Compara Analysis of the Nuclear Hormone Receptors across the selective nematode genomes was performed and used to construct the phylogenetic tree of all the filarial nematodes as well as other Ensembl Compara GeneTree species. The boxes show the Gene IDs of the NHRs duplicated or lost during the evolution of the filarial species. The OVOC35* family consist of 4 NHR genes: OVOC350, OVOC351, OVOC353, and OVOC354.

Supp_Fig.7

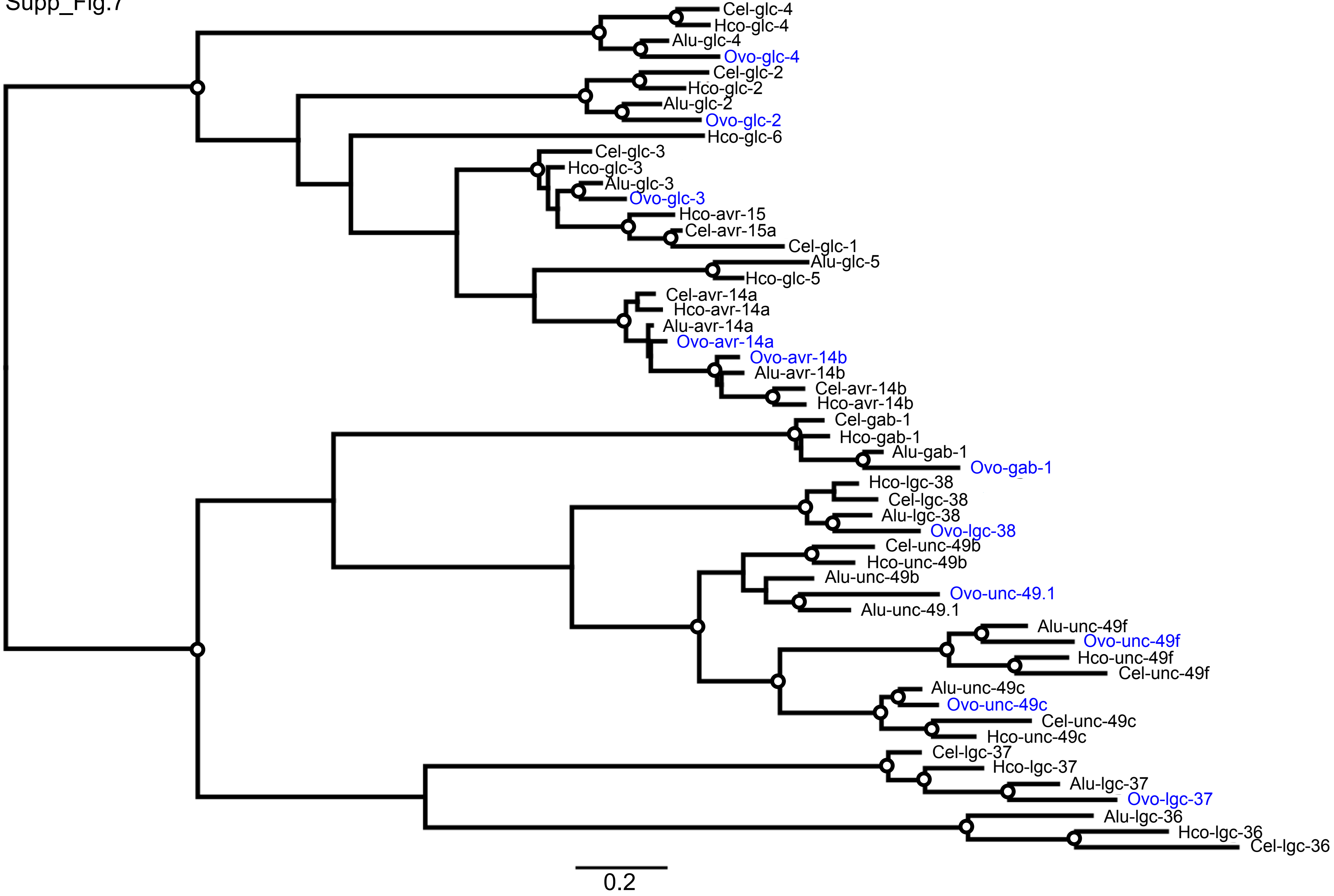
Cel-glc-4
Hco-glc-4
Alu-glc-4
Ovo-glc-4
Cel-glc-2
Hco-glc-2
Alu-glc-2
Ovo-glc-2
Hco-glc-6
Cel-glc-3
Hco-glc-3
Alu-glc-3
Ovo-glc-3
Hco-avr-15
Cel-avr-15a
Cel-glc-1
Alu-glc-5
Hco-glc-5
Cel-avr-14a
Hco-avr-14a
Alu-avr-14a
Ovo-avr-14a
Ovo-avr-14b
Alu-avr-14b
Cel-avr-14b
Hco-avr-14b
Cel-gab-1
Hco-gab-1
Alu-gab-1
Ovo-gab-1
Hco-lgc-38
Cel-lgc-38
Alu-lgc-38
Ovo-lgc-38
Cel-unc-49b
Hco-unc-49b
Alu-unc-49b
Ovo-unc-49.1
Alu-unc-49.1
Alu-unc-49f
Ovo-unc-49f
Hco-unc-49f
Cel-unc-49f
Alu-unc-49c
Ovo-unc-49c
Cel-unc-49c
Hco-unc-49c
Cel-lgc-37
Hco-lgc-37
Alu-lgc-37
Ovo-lgc-37
Alu-lgc-36
Hco-lgc-36
Cel-lgc-36
0.2

Supplementary Figure 7. Nucleotide sequences for pLGIC subunits from the glutamate- and GABA- gated chloride-channel family from *O. volvulus* and the representative species *A. lumbricoides*, *H. contortus* and *C. elegans* were aligned as codons using MAFFT v7.222[1]. Regions corresponding to the signal peptide, the intracellular loop between transmembrane regions 3 and 4 and the C-terminal beyond TM 4 were removed. The phylogeny shown was reconstructed using PhyML v20120412 with white circles indicating nodes with greater than 0.9 SH[2]. The tree is shown rooted by the division between the glutamate- and GABA channel subunits.

Supp_Fig. 8

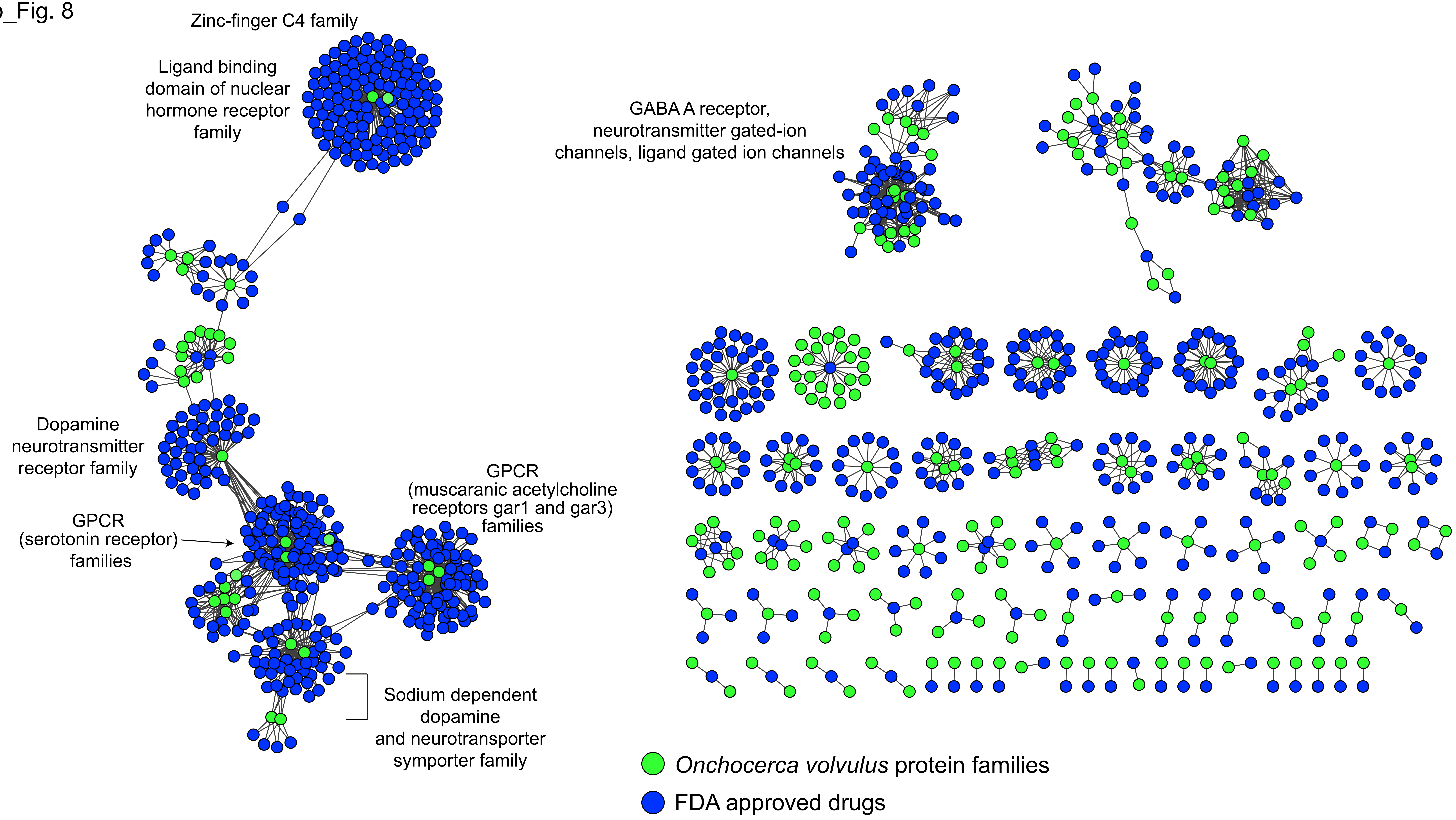
Zinc-finger C4 family
Ligand binding domain of nuclear hormone receptor family
GABA A receptor, neurotransmitter gated-ion channels, ligand gated ion channels
Dopamine neurotransmitter receptor family
GPCR (muscaranic acetylcholine receptors gar1 and gar3) families
GPCR (serotonin receptor) families
Sodium dependent dopamine and neurotransporter symporter family
Onchocerca volvulus protein families
FDA approved drugs

Supplementary Figure 8. *O. volvulus* protein families identified as potential targets of FDA-approved drugs. A network representation of FDA-approved drugs (blue circles) and their potential *O. volvulus* protein family targets (green circles). The predicted drug-protein family target connection is represented by a line connecting the respective circles. Protein families associated with the most drugs are named in the figure and considered 'privileged target families'.

Supplementary Information References

1. Castresana, J. Selection of conserved blocks from multiple alignments for their use in phylogenetic analysis. *Mol Biol Evol* **17**, 540-52 (2000).

2. Guindon, S. *et al.* New algorithms and methods to estimate maximum-likelihood phylogenies: assessing the performance of PhyML 3.0. *Syst Biol* **59**, 307-21 (2010).
